# MiR-335-5p Escaped from CircKIAA0586 Adsorption Contributes to Mechanical Overloading-Induced Cartilage Degeneration by Targeting Lymphoid-Specific Helicase

**DOI:** 10.34133/research.0694

**Published:** 2025-05-08

**Authors:** Haoyu Xie, Yuheng Lu, Jianying Pan, Hua Zeng, Zhicheng Zhang, Jianbin Yin, Jinjian Zhu, Bingsheng Luo, Dong Guo, Chunyu Wu, Chun Zeng, Yan Shao, Xiaochun Bai, Daozhang Cai, Haiyan Zhang

**Affiliations:** ^1^ Department of Joint Surgery, Center for Orthopaedic Surgery, The Third Affiliated Hospital of Southern Medical University, Guangzhou 510630, China.; ^2^ Department of Orthopedics, Orthopedic Hospital of Guangdong Province, Academy of Orthopedics·Guangdong Province, The Third Affiliated Hospital of Southern Medical University, Guangzhou 510630, China.; ^3^ The Third School of Clinical Medicine, Southern Medical University, Guangzhou 510630, China.; ^4^ Guangdong Provincial Key Laboratory of Bone and Joint Degeneration Diseases, Guangzhou 510630, China.; ^5^ Department of Rehabilitation Medicine, Xijing Hospital, Fourth Military Medical University, Xi ’an 710032, China.; ^6^ State Key Laboratory of Organ Failure Research, Department of Cell Biology, School of Basic Medical Sciences, Southern Medical University, Guangzhou 510515, China.

## Abstract

Mechanical overload is a critical contributor to cartilage degeneration in osteoarthritis (OA) pathogenesis. Circular RNA (circRNA) is expected to provide a long-lasting therapy for OA. However, the involvement of the circRNA-associated competitive endogenous RNA network in chondrocyte senescence induced by mechanical overloading remains unestablished. A mechanical overloading-induced chondrocyte senescence model in human primary chondrocytes is constructed, and differences in the expression of circRNAs and miRNAs were analyzed. The biological roles of circKIAA0586/miR-335-5p in chondrocyte senescence and OA progression under mechanical overloading and its downstream targets were determined using gain- and loss-of-function experiments in various biochemical assays in human chondrocytes. The in vivo effects of circKIAA0586 overexpression were also determined in destabilization of the medial meniscus (DMM) OA mice and aged spontaneous OA mice. The mechanical overloading-induced chondrocyte senescence was aggravated by miR-335-5p or circKIAA0586 knockdown. Accumulated DNA damage response was observed following mechanical overloading, which reduced after miR-335-5p inhibition or circKIAA0586 supplementation. MiR-335-5p was regulated by circKIA0586 adsorption. HELLS was prominently down-regulated following mechanical overloading treatment. Moreover, miR-335-5p bound to lymphoid-specific helicase (HELLS) mRNA during mechanical overloading was demonstrated to mediate the nonhomologous end joining (NHEJ) pathway, thereby inducing DNA damage and senescence. In addition, the senescence delaying and cartilage protective functions of circKIAA0586 and HELLS were validated in DMM OA mice and aged spontaneous OA mice. Our findings suggest that miR-335-5p, which escapes circKIAA0586 adsorption, facilitates mechanical overloading-induced chondrocyte senescence and OA progression by impairing the NHEJ pathway through HELLS inhibition. Overall, targeting circKIAA0586/miR-335-5p/HELLS signaling provides a novel therapeutic approach for OA.

## Introduction

Osteoarthritis (OA), a degenerative joint disease that worsens with aging and posttraumatic stress, causes a growing global health challenge particularly affecting individuals over 60 years [1]. As the leading cause of physical disability worldwide, OA places a tremendous economic burden on societies globally [2]. Despite its clinical prevalence, the precise etiology driving OA pathogenesis remains elusive, and no etiological treatment for OA exists [3]. This knowledge gap underscores the critical need for mechanistic studies to develop viable therapeutics for OA.

OA development is considered to be substantially influenced by mechanical overloading [4]. While physiological mechanical loading maintains joint homeostasis [5], supraphysiological stress triggers articular cartilage degradation, initiating OA progression [6]. Clinical observations reveal that most OA patients exhibit a misaligned knee axis, causing further cartilage wear and OA progression [7]. Building on these findings, our prior work using a murine dynamic compression model established a causal relationship between mechanical overload and chondrocyte senescence, which highlights mechano-aging interaction in OA development [8]. Nevertheless, the precise molecular mechanisms underlying mechanical overloading-induced senescence remain incompletely characterized.

Initially regarded as transcriptional "noise" due to their noncoding nature, noncoding RNAs (ncRNAs) including microRNAs (miRNAs), long ncRNAs, and circular RNAs (circRNAs) are now recognized as potent epigenetic regulators that possess powerful gene expression control abilities [9]. Growing evidence implicates these noncoding elements in age-related pathologies [10,11]. Moreover, they are also essential for establishing and maintaining joint homeostatic balance [12]. Notably, ncRNAs have recently emerged as critical mediators of mechanochemical transduction in chondrocytes, orchestrating cellular responses to biomechanical stimuli [13,14]. Emerging evidence suggests that mechanical overloading may drive OA pathogenesis through ncRNA dysregulation, yet mechanistic insights into this mechano-epigenetic interaction remain limited.

miRNA is a family of ncRNAs that typically comprises 18 to 24 nucleotides, which down-regulate gene expression by binding to mRNA, resulting in degradation or suppressing protein translation in diverse diseases [15–17]. In OA pathogenesis, miRNAs critically regulate cartilage matrix catabolism and inflammatory cascades [18,19]. Emerging evidence identifies mechanoresponsive miRNAs that modulate chondrocyte metabolism under mechanical loading [6,14]. Nevertheless, further research is demanded to ascertain the functions of miRNAs in the modulation of chondrocyte aging attributed to mechanical overloading.

Competitive endogenous RNAs (ceRNAs) act as miRNA sponge transcripts and mutually regulate each other by interacting with miRNAs [20]. Among these, circRNAs exhibit unique advantages as natural miRNA sponges due to their covalently closed structures conferring exceptional stability and abundant miRNA binding sites [21]. Recent studies have confirmed that circRNAs regulate chondrocyte senescence and chondrocyte metabolism [22–24]. Notably, several circRNAs have been reported to be mechanically sensitive and involved in regulating chondrocyte function [25,26]. However, the involvement of the circRNA-associated ceRNA network in chondrocyte senescence induced by mechanical overloading remains unestablished.

The nonhomologous end joining (NHEJ) repair pathway is an essential repair for DNA double-strand break (DSB) damage in mammal cells [27]. NHEJ repair occurs when the ring-shaped Ku heterodimer, composed of the Ku80 and Ku70 proteins, identifies and binds to the target DSBs in a sequence-independent manner. HELLS and CDCA7 contribute to NHEJ by facilitating the accumulation of Ku80 at DSBs [28]. Our study reveals that mechanical overloading disrupts NHEJ repair via a novel circKIAA0586/miR-335-5p/HELLS axis. Crucially, we establish that circKIAA0586 depletion mediates mechano-induced senescence through miR-335-5p-dependent impairment of the NHEJ pathway, revealing a previously unrecognized ceRNA network governing mechanical epigenetics in OA pathogenesis.

## Results

### MiR-335-5p plays a pivotal role in mechanical chondrocyte senescence during OA

**Fig. 1.**
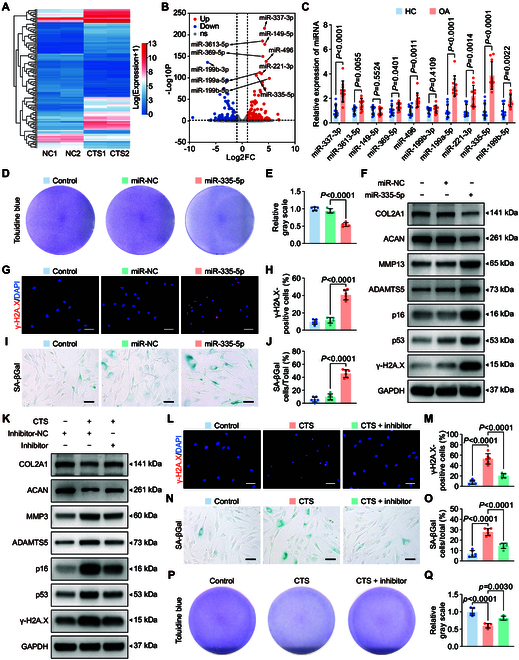
MiR-335-5p plays a pivotal role in mechanical overloading-induced chondrocyte senescence during osteoarthritis (OA). (A and B) Heatmap and volcano plot show differentially expressed miRNAs in overloaded human primary chondrocytes (cyclic tensile strain [CTS]) compared with control cells (NC). The arrows indicate miR-335-5p. (C) Expression of the TOP 10 candidate miRNAs in knee cartilage of OA patients and controls (*n* = 10 samples per group) were confirmed by quantitative reverse transcription polymerase chain reaction (qRT-PCR) analysis. (D and E) Representative images and quantification of toluidine blue staining of controls human primary chondrocytes transfected with miR-335-5p mimic or miR-NC (*n* = 5 per group). (F) Immunoblotting results for COL2A1, MMP13, ACAN, ADAMTS5, senescence markers (p16 and p53), and DNA damage marker γ-H2A.X in controls and human primary chondrocytes transfected with miR-335-5p mimic or miR-negative control (NC). (G to J) Representative images and quantification of γ-H2A.X immunofluorescence staining (G and H) and SA-βGal staining (I and J) in controls and human primary chondrocytes transfected with miR-335-5p mimic or miR-NC (*n* = 6 per group). Scale bars: 40 μm (G) and 20 μm (I). (K) Immunoblotting results for COL2A1, MMP3, ACAN, ADAMTS5, senescence markers (p16 and p53), and DNA damage marker γ-H2A.X in human primary chondrocytes administered with or without 20% CTS loading for 24 h after transfection with miR-335-5p inhibitor or inhibitor-NC. (L to O) Representative images and quantification of γ-H2A.X immunofluorescence staining (L and M) and SA-βGal staining (N and O) in human primary chondrocytes administered with or without 20% CTS loading for 24 h after transfected with miR-335-5p inhibitor or inhibitor-NC (*n* = 6 per group). Scale bars: 40 μm (L) and 20 μm (N). (P and Q) Representative images and quantification of toluidine blue staining of controls and human primary chondrocytes with or without miR-335-5p inhibition after treatment with 20% CTS loading for 24 h (*n* = 5 per group). The statistics are presented as the mean ± SD.

In a previous study, we reported that chondrocyte senescence was simulated by mechanical overloading, exacerbating cartilage degeneration in OA [8]. Nevertheless, the exact mechanisms underlying this pathological process are not completely understood. In this study, miRNA-seq analyses were conducted on human primary chondrocytes exposed to 20% cyclic tensile strain (CTS) loading for 24 h. Compared to the controls, 62 of the 1,766 miRNAs were up-regulated by mechanical overloading (Fig. 1A and B). The top 10 candidate miRNAs with the most obvious differences were further validated using quantitative reverse transcription polymerase chain reaction (qRT-PCR) in clinical cartilage samples. The qRT-PCR results showed that miR-335-5p was the most elevated miRNA in the articular cartilage tissues of patients with OA (Fig. 1C). In addition, the up-regulating effect of mechanical overload on miR-335-5p expression was confirmed in primary human chondrocytes (Fig. S1A). While previous studies implicated miR-335-5p in chondrocyte apoptosis and endochondral ossification [29,30], its mechanoregulation remained unexplored. Our findings first identified miR-335-5p as a mechanical stress-responsive miRNA in chondrocytes. Given its reported pro-senescence activity [31], we prioritized miR-335-5p for further investigation.

Subsequently, we identified the biological functions of miR-335-5p in chondrocytes. MiR-335-5p overexpression in human primary chondrocytes reduced collagen type II alpha 1 chain (COL2A1) and aggrecan (ACAN) expression, whereas a disintegrin and metalloproteinase with thrombospondin motifs 5 (ADAMTS5) and matrix metallopeptidase 13 (MMP13) expression increased, indicating a detrimental effect on chondrogenesis (Fig. 1F and Fig. S1B and D). The pro-catabolic function of miR-335-5p was confirmed by toluidine blue staining, a widely used staining to indicate the abundance of proteoglycans in the cartilage matrix (Fig. 1D and E). Additionally, miR-335-5p overexpression increased the number of cells expressing SA-β-Gal, a well-known senescence indicator, and γ-H2A.X, a DNA damage marker, as well as the expression of p16 and p53 (Fig. 1F to J and Fig. S1D). These findings indicated the pro-catabolic and pro-senescent functions of miR-335-5p in human primary chondrocytes, which is consistent with the results of excessive mechanical loading in chondrocytes, as shown in the previous study [8]. Furthermore, it was observed that miR-335-5p silencing partially mitigated the induction of catabolic and senescent effects caused by excessive mechanical loading (Fig. 1K to Q and Fig. S1C and E). These findings collectively indicate that miR-335-5p acts as a negative regulator of chondrocyte rejuvenation and function in response to mechanical overloading.

### MiR-335-5p promotes chondrocyte senescence by inhibiting HELLS

**Fig. 2.**
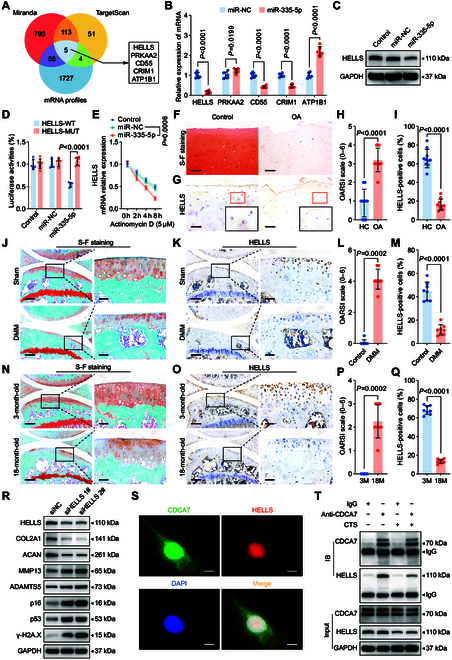
MiR-335-5p promotes chondrocyte senescence by inhibiting HELLS. (A) Schematic illustration showing the overlap of the target mRNAs of miR-335-5p, as predicted by Miranda, TargetScan, and mRNAs down-regulated in human chondrocytes treated with 20% CTS loading for 24 h. (B) qRT-PCR analysis detected target mRNA levels in human chondrocytes transfected with miR-335-5p (*n* = 6 per group). (C) Immunoblotting results for HELLS in control and human primary chondrocytes transfected with miR-NC or miR-335-5p. (D) Luciferase reporter assay showing luciferase activity of HELLS-wild type (WT) or HELLS-mutant (MUT) reporter plasmids in HEK-293T cells transfected with miR-335-5p or miR-NC (*n* = 6 per group). (E) qRT-PCR analysis detected the degradation of HELLS mRNA in human primary chondrocytes transfected with miR-335-5p or miR-NC after treated with 5 μmol·l^−1^ Actinomycin D at 0, 2, 4, and 8 h (*n* = 3 per time point). (F to I) Representative images and quantification of safranin O and fast green staining (F and G) and HELLS immunohistochemical staining (H and I) in knee cartilage of control and OA patients (*n* = 10 samples per group). Scale bar: 100 μm. (J to M) Representative images and quantification of safranin O and fast green staining (J and L) and immunohistochemical staining of HELLS (K and M) in knee cartilage from sham and destabilization of the medial meniscus (DMM) mice (*n* = 8 per group). Scale bars: 200 μm and 40 μm. (N to Q) Representative images and quantification of safranin O and fast green staining (N and P) and immunohistochemical staining of HELLS (O and Q) in knee cartilage from 3-month-old mice and 18-month-old spontaneous OA mice (*n* = 8 per group). Scale bars: 200 μm and 40 μm. (R) Immunoblotting results for HELLS, COL2A1, MMP13, ACAN, ADAMTS5, p16, p53, and γ-H2A.X in human primary chondrocytes after transfection with HELLS siRNA (siHELLS) or siNC. (S) Colocalization of CDCA7 and HELLS using immunofluorescent staining of human primary chondrocytes. Scale bar: 10 μm. (T) Coimmunoprecipitation assay using CDCA7 as bait protein in human primary chondrocytes exposed to or not to 20% CTS loading for 24 h. All chondrocytes from the surface of cartilage to the boundaries between cartilage and subchondral bone were included in the count. Data are presented as mean ± SD.

Potential miR-335-5p targets were identified by comparing RNA-seq analysis results with the predicted outcomes of TargetScan and Miranda to understand how miR-335-5p promotes chondrocyte senescence during OA development (Fig. 2A). Among 5 potential targets, qRT-PCR demonstrated that the miR-335-5p mimics down-regulated 3 genes and HELLS is the most obviously down-regulated gene (Fig. 2B). HELLS is essential for repairing DNA damage, specifically DSB repair [32]. Compared with the other targets, HELLS is closely related to aging since its deletion has been linked to premature aging in mice models [33]. Therefore, HELLS was selected as the target of miR-335-5p for further investigation. The reduction in HELLS by miR-335-5p was confirmed using an immunoblot assay at the protein level (Fig. 2C). HELLS shared a conserved coding sequence binding site for miR-335-5p (Fig. S2A). The luciferase assay established that miR-335-5p binds to HELLS, and this interaction was further determined using an RNA stability assay, demonstrating that miR-335-5p impairs HELLS mRNA stability (Fig. 2D and E).

**Fig. 3.**
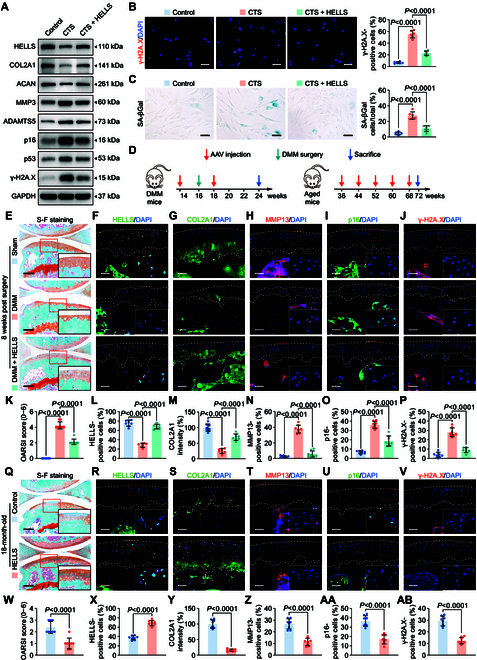
HELLS alleviates chondrocyte senescence and OA progression. (A) Immunoblotting results for HELLS, COL2A1, MMP3, ACAN, ADAMTS5, senescence markers (p16 and p53), and DNA damage marker γ-H2A.X in controls and human primary chondrocytes with or without HELLS overexpression after treatment with 20% CTS loading for 24 h. (B and C) Representative images and quantification of γ-H2A.X immunofluorescence staining (B) and SA-βGal staining (C) in controls and human primary chondrocytes with or without HELLS overexpression after treated with 20% CTS loading for 24 h (*n* = 6 per group). Scale bars: 40 μm (B) and 20 μm (C). (D) Schematic illustration showing the establishment of the DMM OA mouse model and aged spontaneous OA mouse model. DMM surgery is shown by the green arrows. The red arrows indicate the AAV intra-articular injection time points. The blue arrows indicate the sacrifice time points. (E to P) Representative images and quantification of safranin O and fast green staining (E and K) and HELLS (F and L), COL2A1 (G and M), MMP13 (H and N), p16 (I and O) and γ-H2A.X (J and P) immunofluorescence staining in knee cartilage from sham mice and DMM mice with control AAV or HELLS AAV administered at 8 weeks after DMM surgery (*n* = 8 per group). Scale bars: 100 μm (E) and 40 μm (F to J). (Q to AB) Representative images and quantification of safranin O and fast green staining (Q and W) and HELLS (R and X), COL2A1 (S and Y), MMP13 (T and Z), p16 (U and AA), and γ-H2A.X (V and AB) immunofluorescence staining in knee cartilage from aged spontaneous OA mice with control AAV or HELLS AAV administered (*n* = 8 per group). Scale bars: 100 μm (Q) and 40 μm (R to V). All chondrocytes from the surface of cartilage (white dotted line on top) to the boundaries between cartilage and subchondral bone (white dotted line below) were included in the count. The statistics are shown as the mean ± SD.

Subsequently, we examined the biological functions of HELLS in chondrocytes. HELLS levels were decreased in response to mechanical overloading in chondrocytes and were reduced in the articular cartilage tissues of aged spontaneous OA mice, mechanical loading-induced destabilization of the medial meniscus (DMM) OA mice, and OA patients (Fig. 2F to Q and Fig. S2B to E). Silencing HELLS in chondrocytes obviously down-regulated COL2A1 and ACAN expression, up-regulated MMP13 and ADAMTS5 expression, and increased p16 and p53 expression (Fig. 2R and Fig. S3A). Staining of γ-H2A.X and SA-βGal demonstrated substantial up-regulation (Fig. S3D to G), while toluidine blue staining became lighter after knockdown of HELLS (Fig. S3B and C). Moreover, supplementation with HELLS reversed the pro-senescent and pro-catabolic effects of mechanical overloading in vitro (Fig. 3A to C and Fig. S4A to C) and inhibited cartilage degeneration in DMM mice (Fig. 3E to P) and aged spontaneous OA mice (Fig. 3Q to AB). We introduced a spontaneous OA model here to further investigate whether overexpression of HELLS could inhibit the natural progression of OA. Intra-articular injections of HELLS adeno-associated virus (AAV) were administered 2 weeks before and after DMM surgery for DMM mice (Fig. 3D). In the aged spontaneous OA mouse model, mice were treated with intra-articular injections every 2 months, from 9 months to 18 months old (Fig. 3D). Considering the attenuation of the overexpression effect by AAV, we performed a series of injections and attempted to achieve a sustained overexpression effect. According to the manufacturer ’s protocol, the peak expression of AAV occurred 3–4 weeks after injection and the expression decreased to half at 8–12 months. Therefore, single-dose AAV cannot maintain a stable expression effect in a 9-month period. Consequently, we performed the injection every 8 weeks.

Furthermore, we determined the mechanism by which HELLS controls chondrocyte senescence. Given that HELLS is essential for DNA damage repair via NHEJ pathway by interacting with CDCA7 [28], we hypothesized that chondrocyte senescence caused by mechanical overloading resulted from the reduced interaction of HELLS and CDCA7. Immunofluorescence showed abundant CDCA7 in normal chondrocytes and its colocalization with HELLS in the nucleus (Fig. 2S). Furthermore, decreased binding of HELLS and CDCA7 during mechanical overloading was confirmed using a co-immunoprecipitation (Co-IP) assay (Fig. 2T). These findings suggest that HELLS is suppressed by miR-335-5p, thus accelerating chondrocyte senescence.

### HELLS with synonymous mutation for miR-335-5p results in protection against OA

**Fig. 4.**
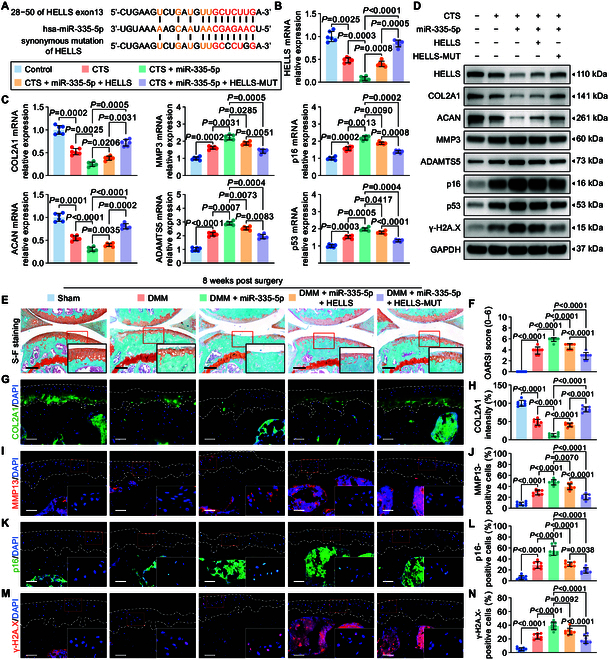
HELLS with synonymous mutation for miR-335-5p results in protection against OA. (A) Schematic illustration to show the synonymous mutation in the binding site of HELLS mRNA for miR-335-5p. (B and C) qRT-PCR analysis of HELLS (B), COL2A1, MMP3, ACAN, ADAMTS5, and senescence markers (p16 and p53) in human primary chondrocytes treated with 20% CTS loading for 24 h after transfection with miR-335-5p alone or together with HELLS or HELLS-MUT plasmids (*n* = 6 per group). (D) Immunoblotting results for COL2A1, MMP3, ACAN, ADAMTS5, senescence markers (p16 and p53), and DNA damage marker γ-H2A.X in controls and human primary chondrocytes treated with 20% CTS loading for 24 h after transfection with miR-335-5p alone or together with HELLS or HELLS-MUT plasmids. (E to J) Representative images and quantification of safranin O and fast green staining (E and F) and COL2A1 (G and H), MMP13 (I and J), p16 (K and L), and γ-H2A.X (M and N) immunofluorescence staining in knee cartilage from sham mice and DMM mice administered miR-335-5p AAV alone or together with HELLS AAV or HELLS-MUT AAV at 8 weeks after DMM surgery (*n* = 8 per group). Scale bars: 100 μm (E) and 40 μm (G, I, K, and M). All chondrocytes from the surface of cartilage (white dotted line on top) to the boundaries between cartilage and subchondral bone (white dotted line below) were included in the count. The statistics are presented as the mean ± SD.

A miR-335-5p mimic alone or together with HELLS or HELLS-mutant (MUT, synonymous mutation in the binding site of HELLS mRNA for miR-335-5p, as shown in Fig. 4A) was transfected into mechanical overloading-treated chondrocytes to determine whether miR-335-5p participates in mechanical overloading-induced OA by targeting HELLS. The pro-senescent and pro-catabolic effects of mechanical overloading led to an up-regulation of miR-335-5p, which was effectively reversed more by HELLS-MUT than by HELLS in chondrocytes. This suggests that miR-335-5p affects chondrocytes by targeting HELLS (Fig. 4B to D and Fig. S5A to C).

To further investigate whether miR-335-5p affects chondrocyte senescence and cartilage degeneration by targeting HELLS, we employed AAV miR-335-5p, which was intra-articularly injected into aged spontaneous OA and DMM mice with HELLS AAV or HELLS-MUT AAV. Intra-articular injections were administered as mentioned above (Fig. 3D). The up-regulation of miR-335-5p and HELLS was validated through fluorescence in situ hybridization (FISH) and immunofluorescence assays (Fig. S6A to H). As anticipated, the supplementary administration of miR-335-5p exacerbated the progression of OA development, manifesting as aggravated cartilage degeneration and proteoglycan loss, increased MMP13 expression, and reduced COL2A1 expression in the mouse tibial cartilage (Fig. 4E to J and Fig. S6I to K, N, and O). Importantly, p16-positive and γ-H2A.X-positive chondrocytes were substantially increased following miR-335-5p treatment (Fig. 4K to N and Fig. S6L, M, P, and Q). Moreover, the injection of HELLS-MUT AAV, rather than HELLS AAV, mitigated the degenerative changes in cartilage induced by miR-335-5p, such as increased catabolic response, decreased extracellular matrix (ECM) composition, and enhanced senescence and DNA damage in both DMM and aged spontaneous OA models (Fig. 4E to N and Fig. S6I to Q). Collectively, these results indicated that miR-335-5p accelerates chondrocyte senescence and cartilage degeneration by suppressing HELLS both in vivo and in vitro.

## MiR-335-5p is sponged by circKIAA0586

**Fig. 5.**
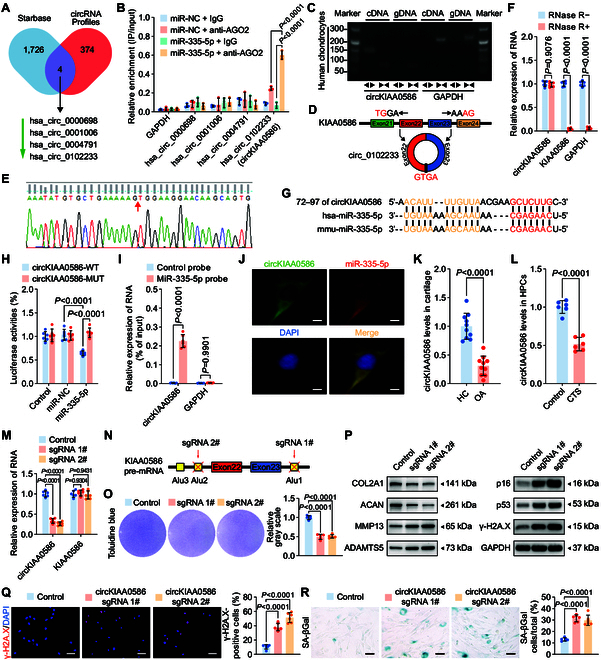
CircKIAA0586 sponges miR-335-5p to regulate chondrocyte senescence and metabolism. (A) Schematic representation demonstrating the overlap of the upstream circRNAs of miR-335-5p, as predicted by TargetScan and circRNAs down-regulated in human chondrocytes treated with 20% CTS loading for 24 h. (B) AGO2 RNA immunoprecipitation in SW1353 cells transfected with miR-335-5p or miR-NC. The candidate circRNAs and GAPDH levels were quantified by qRT-PCR analysis, and the relative IP-to-input ratios were plotted (*n* = 3 per group). (C to E) Agarose gel electrophoresis (C) shows that divergent primers (←→) amplified circKIAA0586 in complementary DNA (cDNA) but not genomic DNA (gDNA) (top). The amplified product of specific divergent primers was confirmed in keeping with the sequence (D) of circKIAA0586 validated using Sanger sequencing (E). (F) qRT-PCR analysis for the abundance of circKIAA0586, KIAA0586 mRNA, and GAPDH mRNA in human primary chondrocytes treated with or without RNase R (*n* = 6 per group). (G) Schematic illustration to show the conserved circKIAA0586 binding site of human and mouse miR-335-5p. (H) Luciferase reporter assay shows luciferase activity of circKIAA0586-WT or -MUT reporter plasmids in controls and HEK-293T cells transfected with miR-335-5p or miR-NC (*n* = 6 per group). (I) RNA pulldown assay in SW1353 cells transfected with biotinylated miR-335-5p probes or control probes. The levels of circKIAA0586 and GAPDH were quantified using qRT-PCR analysis, and relative levels of circKIAA0586 were normalized to the input levels (*n* = 6 per group). (J) Representative FISH images showing the colocalization of circKIAA0586 and miR-335-5p in human primary chondrocytes. Scale bar: 10 μm. Data are presented as the mean ± SD. (K) qRT-PCR analysis detected circKIAA0586 levels in knee cartilage of controls and OA patients (*n* = 10 samples per group). (L) qRT-PCR analysis detected circKIAA0586 levels in human primary chondrocytes administered with 20% CTS loading for 24 h and control cells (*n* = 6 per group). (M and N) qRT-PCR analysis (M) validated the specific knockdown of circKIAA0586 in human primary chondrocytes after transfection of CRISPR/Cas9 knockdown plasmids targeting circKIAA0586 cyclization elements (N) (*n* = 6 per group). (O) Representative images and quantification of toluidine blue staining of human primary chondrocytes transfected with circKIAA0586 knockdown plasmids or control empty vector (*n* = 5 per group). (P) Immunoblotting results for OL2A1, MMP13, ACAN, ADAMTS5, senescence markers (p16 and p53), and DNA damage marker γ-H2A.X in human primary chondrocytes with circKIAA0586 knockdown plasmids or control plasmids. (Q and R) Representative images and quantification of γ-H2A.X immunofluorescence staining (Q) and SA-βGal staining (R) in human primary chondrocytes transfected with circKIAA0586 knockdown plasmids or control empty vector (*n* = 6 per group). Scale bars: 40 μm (Q) and 20 μm (R). Data are presented as the mean ± SD.

As predicted using StarBase analysis, miR-335-5p has multiple binding sites for several circRNAs. By overlapping the predicted outcomes of StarBase and the circRNA sequences, we identified 4 potential circRNAs (Fig. 5A). Among these, circKIAA0586 was selected for further investigation due to its highest abundance among the 4 circRNAs when isolated from SW1353 cells using argonaute RNA-induced silencing complex catalytic component 2 (AGO2) immunoprecipitation (Fig. 5B). Subsequently, qRT-PCR assays determined the presence of circKIAA0586 in the cartilage using specific divergent primers (Fig. 5C). The predicted splice junction of circKIAA0586 (Fig. 5D) was confirmed in cartilage tissues. The amplified product obtained using divergent primers was identical to the circKIAA0586 sequence obtained by Sanger sequencing (Fig. 5E). Notably, circKIAA0586 exhibited resistance to RNase R, while the level of KIAA0586 mRNA markedly decreased following RNase R treatment (Fig. 5F).

To determine whether circKIAA0586 binds to miR-335-5p, we compared the sequences of circKIAA0586 and miR-335-5p and identified a conserved circKIAA0586 binding site for miR-335-5p (Fig. 5G). This binding was confirmed using a luciferase assay (Fig. 5H). Furthermore, a pulldown assay showed that circKIAA0586 was pulled down by the miR-335-5p probes when compared with the control probes (Fig. 5I). Additionally, primary chondrocytes displayed colocalization of circKIAA0586 and miR-335-5p in the cytoplasm, as demonstrated by RNA FISH (Fig. 5J). These findings collectively indicated that circKIAA0586 directly binds to miR-335-5p in chondrocytes.

### CircKIAA0586 exhibits relatively low levels in OA cartilage and regulates chondrocyte senescence and metabolism

We assessed circKIAA0586 expression during the development of OA. qRT-PCR analysis revealed a substantial decrease in circKIAA0586 expression in human OA cartilage (Fig. 5K). Moreover, mechanical overloading down-regulated circKIAA0586 in human primary chondrocytes when compared with that in the controls (Fig. 5L). To explore the role of circKIAA0586 in OA progression during mechanical overloading, circKIAA0586 knockdown chondrocytes using the CRISPR/Cas9 gene-editing system were generated, targeting the circKIAA0586 cyclization elements (Fig. 5N). qRT-PCR showed that transfection of the circKIAA0586 knockdown vector led to the decreased expression of circKIAA0586 in chondrocytes but did not change KIAA0586 mRNA levels (Fig. 5M). Loss of circKIAA0586 resulted in a higher proportion of senescent cells; increased γ-H2A.X activation; lighter toluidine blue staining; elevated expression of p16, p53, ADAMTS5, and MMP13; and reduced expression of ACAN and COL2A1 (Fig. 5O to R and Fig. S7A). Thus, these findings indicate that circKIAA0586 silencing leads to chondrocyte senescence and cartilage degradation.

### CircKIAA0586 alleviates OA by adsorbing miR-335-5p

**Fig. 6.**
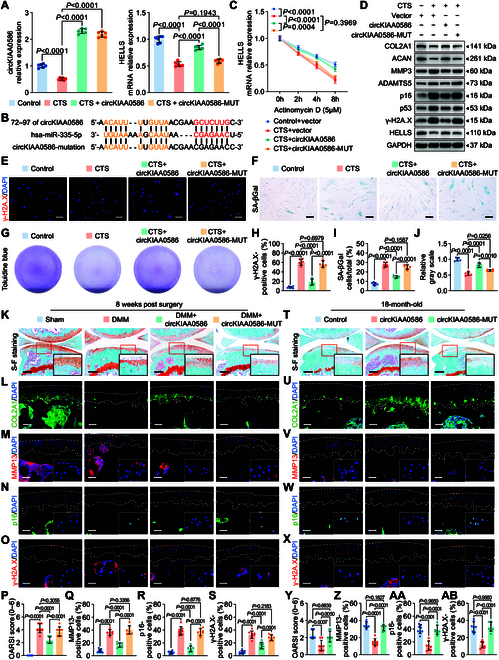
CircKIAA0586 alleviates OA by adsorbing miR-335-5p. (A) qRT-PCR analysis of circKIAA0586 and HELLS in human primary chondrocytes exposed to 20% CTS loading for 24 h after transfection of control vector, circKIAA0586, or circKIAA0586-MUT (*n* = 6 per group). (B) Schematic depiction to demonstrate the mutation in the binding site of circKIAA0586 for miR-335-5p. (C) qRT-PCR analysis detected the degradation of HELLS mRNA in human primary chondrocytes overexpressing control vector, circKIAA0586, or circKIAA0586-MUT after treating with 20% CTS loading and 5 μmol·l^−1^ Actinomycin D together at 0, 2, 4, and 8 h (*n* = 3 per time point). (D) Immunoblotting results for HELLS, COL2A1, MMP3, ACAN, ADAMTS5, p16, p53, and γ-H2A.X in human primary chondrocytes treated with 20% CTS loading for 24 h after transfection of control vector, circKIAA0586, or circKIAA0586-MUT. (E to J) Representative images and quantification of toluidine blue staining (E and H), γ-H2A.X immunofluorescence staining (F and I), and SA-βGal staining (G and J) in human primary chondrocytes treated with 20% CTS loading for 24 h after transfection of control vector, circKIAA0586, or circKIAA0586-MUT (*n* = 5 or 6 per group). Scale bars: 40 μm (E) and 20 μm (F). (K to S) Representative images and quantification of safranin O and fast green staining (K and P) and COL2A1 (L), MMP13 (M and R), p16 (N and R), and γ-H2A.X (O and S) immunofluorescence staining in knee cartilage from sham and DMM mice administered control AAV, circKIAA0586 AAV, or circKIAA0586-MUT AAV at 8 weeks after DMM surgery (*n* = 8 per group). Scale bars: 100 μm (K) and 40 μm (L to O). (T to AB) Representative images and quantification of safranin O and fast green staining (T and Y) and COL2A1 (U), MMP13 (V and Z), p16 (W and AA), and γ-H2A.X (X and AB) immunofluorescence staining in knee cartilage from 18-month-old mice administered control AAV, circKIAA0586 AAV, or circKIAA0586-MUT AAV (*n* = 8 per group). Scale bars: 100 μm (first row) and 40 μm (other rows). All chondrocytes from the surface of cartilage (white dotted line on top) to the boundaries between cartilage and subchondral bone (white dotted line below) were included in the count. Data are presented as means ± SD.

We investigated whether circKIAA0586 regulates OA development by interacting with miR-335-5p. The reduction in circKIAA0586 expression in chondrocytes subjected to excessive mechanical loading was reversed by supplementation with circKIAA0586 or circKIAA0586-MUT (disruption of the binding site of circKIAA0586 for miR-335-5p, shown in Fig. 6B) (Fig. 6A). However, circKIAA0586, not circKIAA0586-MUT, reduced miR-335-5p expression, suppressed the pro-senescent and pro-catabolic effects of mechanical overloading, and restored COL2A1 and ACAN expression (Fig. 6D to H and Fig. S8A). Moreover, circKIAA0586 reinstated the inhibition of HELLS and stabilized HELLS mRNA expression during excessive mechanical loading (Fig. 6A and C).

In accordance with the findings of the animal study, the protective role of circKIAA0586 during OA development was observed in DMM and aged mice with spontaneous OA. FISH and immunohistochemical staining revealed that treatment with circKIAA0586 inhibited miR-335-5p expression and increased HELLS expression, whereas circKIAA0586-MUT did not produce the same effect (Fig. S9A to L). As expected, only supplementary administration of circKIAA0586 effectively ameliorated OA progression in mice, as demonstrated by reduced cartilage damage and proteoglycan loss, decreased MMP13 expression, and increased COL2A1 expression in the tibial cartilage (Fig. 6K to M, P, and Q and Fig. 6T to V, Y, and Z). Meanwhile, p16-positive and γ-H2A.X-positive chondrocytes were markedly reduced by circKIAA0586 but not by circKIAA0586-MUT (Fig. 6N, O, R, and S and Fig. 6W to X, AA, and AB). These findings collectively suggest that elevated circKIAA0586 expression protects against OA by targeting miR-335-5p and HELLS.

## Discussion

Both aging and abnormal mechanical stress substantially contribute to OA development [4,34]. Our study delineates a novel mechano-epigenetic axis centered on the circKIAA0586/miR-335-5p/HELLS network, revealing how mechanical overloading disrupts DNA repair fidelity to promote chondrocyte senescence. Mechanical stress down-regulates the miRNA sponge circKIAA0586, enabling the accumulation of miR-335-5p, which targets HELLS mRNA. This impairs the ability of HELLS/CDCA7 complex to recruit Ku80 for NHEJ-mediated DNA double-strand break repair, ultimately leading to chondrocyte senescence and cartilage degeneration. Therapeutic restoration of circKIAA0586 or HELLS expression demonstrated obvious disease-modifying effects across preclinical models.

Initially characterized as a tumor metastasis suppressor, miR-335-5p, encoded by the second intron of the MEST gene, is known to target tenascin C and Sry-box transcription factor 4 [31]. Previous research has reported its role in promoting endothelial cell senescence by targeting SIRT7 and sKlotho, both genes associated with senescence [35,36], as well as its reduction of DKK1, an inhibitory factor of the wnt/β-catenin signaling pathway critical in OA development [37]. Recent research has also confirmed that miR-335-5p inhibits HBP1, promoting chondrocyte apoptosis in vitro [29]. However, previous studies have not fully elucidated its role in chondrocyte senescence, and in vivo experimental evidence is lacking. Our study uncovered that miR-335-5p expression was remarkably increased in the chondrocytes in response to excessive mechanical loading. Furthermore, miR-335-5p overexpression in chondrocytes leads to chondrocyte senescence and exacerbates OA. In addition, suppression of miR-335-5p partly counteracted the pro-senescent and pro-catabolic effects of mechanical overload in chondrocytes. Our findings propose an innovative mechanism for chondrocyte senescence in OA that miR-335-5p expression is increased in cartilage chondrocytes due to mechanical overloading. Furthermore, as a natural carrier, exosomes exhibit low immunogenicity and high biocompatibility, which can protect miRNA from degradation and improve treatment efficiency. Consequently, producing miR-335-5p-rich exosomes for the treatment of OA is a promising therapeutic approach.

In this study, the chondrocytes subjected to excessive CTS induced a range of pathogenic responses, which were consistent with a previous study [8]. Our results aligned with previous research showing that excessive CTS stimulation of chondrocytes resulted in a pattern of alterations comparable to those seen in OA patients or animal models. These alterations included the accumulation of DNA damage, accelerated senescence, increased expression of ECM degradation enzymes, and decreased expression of cartilage matrix proteins (type 2 collagen and aggrecan), while supplementation with HELLS exhibited anti-senescent and ECM protective abilities against mechanical overloading. Additionally, a synonymous mutation in HELLS mRNA prevented miR-335-5p from binding to its site, thus reversing its inhibitory effect. Furthermore, bioinformatics investigations revealed that HELLS have a conserved CDS-binding site for miR-335-5p across different species, and the attachment of miR-335-5p to HELLS mRNA weakened the durability of HELLS mRNA. Overall, these findings confirmed that miR-335-5p specifically targets HELLS, resulting in a decrease in the pro-senescent consequences of mechanical overloading in chondrocytes.

As chromatin remodelers, HELLS belongs to the SNF2 helicase protein family. Several epigenetic processes are regulated by HELLS, including nucleosome remodeling, DNA methylation, heterochromatin formation, and histone modifications [38–41]. HELLS is also crucial for the repair of DNA damage, especially DSBs, through its interactions with CDCA7 and CtIP to facilitate NHEJ and homologous recombination [28,42]. Moreover, HELLS is essential for the development of organisms, as it is highly expressed in embryonic stem cells, germ cells, skin, and lymphoid tissue cells [43]. Mutations in the HELLS gene have been reported to be associated with the human immunodeficiency syndrome ICF (immunodeficiency, centromeric instability, facial anomalies) [44]. In recent studies, HELLS has been linked to several types of cancers [45–48]. However, its contribution to chondrocyte senescence and the onset of OA has not received much attention. Our results corroborated that HELLS expression was reduced in chondrocytes after mechanical overload. HELLS knockdown in chondrocytes results in DNA damage and senescence accumulation in vitro. In addition, senescent responses were inhibited by exogenous supplementation with HELLS in chondrocytes during mechanical overloading and in OA mice. Moreover, weakened interactions between HELLS and CDCA7 were detected in chondrocytes during mechanical overloading, which led to impaired NHEJ and may thus account for the accumulation of DNA damage and chondrocyte senescence induced by mechanical overloading.

As chromatin remodelers, HELLS belongs to the SNF2 helicase protein family. Several epigenetic processes are regulated by HELLS, including nucleosome remodeling, DNA methylation, heterochromatin formation, and histone modifications [38–41]. HELLS is also crucial for the repair of DNA damage, especially DSBs, through its interactions with CDCA7 and CtIP to facilitate NHEJ and homologous recombination [28,42]. Moreover, HELLS is essential for the development of organisms, as it is highly expressed in embryonic stem cells, germ cells, skin, and lymphoid tissue cells [43]. Mutations in the HELLS gene have been reported to be associated with the human immunodeficiency syndrome ICF (immunodeficiency, centromeric instability, facial anomalies) [44]. In recent studies, HELLS has been linked to several types of cancers [45–48]. However, its contribution to chondrocyte senescence and the onset of OA has not received much attention. Our results corroborated that HELLS expression was reduced in chondrocytes after mechanical overload. HELLS knockdown in chondrocytes results in DNA damage and senescence accumulation in vitro. In addition, senescent responses were inhibited by exogenous supplementation with HELLS in chondrocytes during mechanical overloading and in OA mice. Moreover, weakened interactions between HELLS and CDCA7 were detected in chondrocytes during mechanical overloading, which led to impaired NHEJ and may thus account for the accumulation of DNA damage and chondrocyte senescence induced by mechanical overloading.

To identify miR-335-5p upstream signaling during chondrocyte senescence and OA development, we investigated circKIAA0586 (circ_0102233), a novel circRNA generated by the reverse splicing of exons 22 and 23 of KIAA0586. As the parent gene for circKIAA0586, the KIAA0586 gene is located at the 14q23.1 locus [49], encoding a conserved centrosomal protein critical for ciliogenesis [50]. While the precise roles of most circRNAs are not fully understood, accumulating evidence supports their function as miRNA sponges [51]. In this study, qRT-PCR and FISH assays revealed elevated levels of circKIAA0586 in chondrocytes. Furthermore, bioinformatic analyses and molecular biology experiments confirmed a target site for miR-335-5p within circKIAA0586 (conserved between humans and mice). Notably, circKIAA0586 effectively inhibited the expression and biological activity of miR-335-5p. The regulatory effect of circKIAA0586 on miR-335-5p was nullified by its mutation, resulting in the loss of its anti-senescent and anticatabolic properties in chondrocytes subjected to mechanical overload. These findings collectively suggest that circKIAA0586 is a sponge for miR-335-5p, which helps maintain a balance between DNA damage and repair, ultimately delaying chondrocyte senescence and developing spontaneous or surgical instability in OA.

The limitations of this study include that beyond the ceRNA regulatory mechanism, other potential mechanisms of circKIAA0586 might exist, mediating chondrocyte senescence in response to mechanical overloading during OA development. Further investigations are required to understand the role of circKIAA0586 in OA progression. Moreover, since synovitis and abnormal subchondral remodeling are also pivotal causes of OA, future research should comprehensively investigate the involvement of circKIAA0586/miR-335-5p in the synovium or subchondral bone region rather than solely focusing on the cartilage. Finally, although intra-articular AAV injection is a widely used method to infect chondrocytes in OA research, potential risks of AAV diffusion to the meniscus, ligament, synovium, and other tissues in the joint cavity exist. Therefore, more precise methods of molecular delivery to chondrocytes in vivo should be developed to facilitate a better understanding of the functions and underlying mechanisms of circKIAA0586 in OA.

**Fig. 7.**
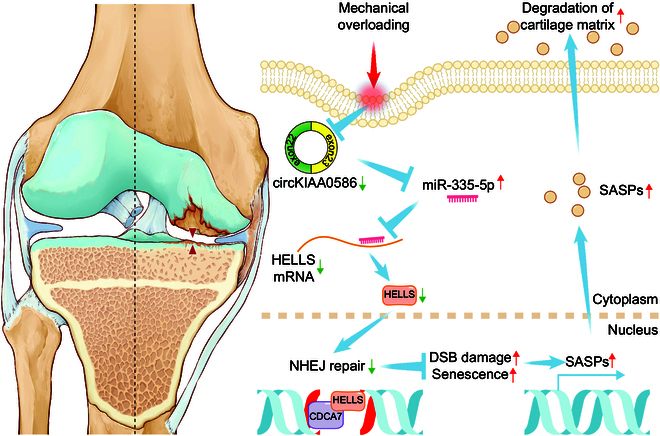
Schematic representation of the working hypothesis. During mechanical overloading, miR-335-5p evades the adsorption of circKIAA0586, allowing it to bind with HELLS mRNA and suppress HELLS expression. Consequently, loss of HELLS impairs the DNA damage repair function of the HELLS/CDCA7 complex, leading to DNA damage accumulation and chondrocyte senescence, resulting in accelerated cartilage degeneration and OA development.

Summarily, miR-335-5p evades circKIAA0586 adsorption and facilitates mechanical overloading-induced chondrocyte senescence and ECM degeneration by targeting HELLS (Fig. 7). Thus, our findings provide novel insights into the identification of key circRNA-associated ceRNA network–mechanics interactions in chondrocyte senescence and OA development. A novel approach for OA treatment can be developed by targeting the circKIAA0586/miR-335-5p/HELLS signaling.

## Materials and Methods

The detailed methods are shown in the Supplementary Materials.

### Clinical cartilage sample collection

During total knee arthroplasty, cartilage samples were collected from the tibial plateaus and femoral condyle of patients with OA (*n* = 10). The rough areas were classified as lesioned cartilage, whereas the smooth areas were classified as normal cartilage. Table S1 provides a summary of the comprehensive patient information. Human cartilage samples were obtained from the Third Affiliated Hospital of Southern Medical University (Guangzhou, China). Before utilizing their clinical data for scientific investigation, all patients provided informed consent. This study was approved and supervised by the Ethics Committee of the Third Affiliated Hospital of Southern Medical University (Guangzhou, China) (approval no. 2022-lunshen-053).

### Animals

From the Experimental Animal Center of the Southern Medical University (Guangzhou, China), 96 male C57BL/6J mice at the age of 8 weeks were obtained. Mice were raised until 16 weeks of age. Our previous research describes the method of DMM model surgery on the right knees of mice [52]. Briefly, the mice were anesthetized with 1% pentobarbital (50 mg·kg^−1^ body weight), and the medial meniscotibial ligament was incised after exposing the right knee joint capsule medially to the patellar tendon. After the assessment, the medial meniscus, right knee joint capsule, and skin were stitched. A simulated surgical procedure was performed on the right knee. The joint capsule was opened and closed without the intervention of the meniscus. For the investigation of the function of circKIAA0586 in experimental OA, 32 mice were randomly assigned to the following 4 groups: sham, control DMM, DMM + circKIAA0586, and DMM + circKIAA0586-MUT groups. For the assessment of the function of HELLS in experimental OA, 24 mice were randomly divided into 3 groups: sham, control DMM, and DMM + HELLS groups. Furthermore, 40 mice were divided into 5 groups, including sham, control DMM, DMM + miR-335-5p, DMM + miR-335-5p + HELLS, and DMM + miR-335-5p + HELLS-MUT groups, to investigate the function of miR-335-5p in OA. Each group contained 8 mice. Geneseed (Guangzhou, China) constructed an AAV containing circKIAA0586 or circKIAA0586-MUT, which was packaged by Ubigene Biosciences Co., Ltd. (Guangzhou, China) along with a control AAV. AAVs containing HELLS or HELLS-MUT, as well as a control AAV, were produced and packaged by HanBio (Shanghai, China). GenePharma (Shanghai, China) supplied both the AAV that overexpressed miR-335-5p and the corresponding control AAV. The mice in each group received AAV intra-articular injection 2 weeks before and after DMM surgery. Mice in the control groups received control AAV for the same period. The right legs were collected after an 8-week postoperative period.

To create a spontaneous OA mouse model, 72 male C57BL/6J mice were obtained from the Experimental Animal Centre of Southern Medical University (Guangzhou, China) when they were 36 weeks old. Notably, mice were raised until 72 weeks of age. Three groups (control, circKIAA0586, and circKIAA0586-MUT groups) were formed by randomly dividing 24 mice to investigate the function of circKIAA0586 in spontaneous OA. In particular, 16 mice were randomly divided into the control and HELLS groups to investigate the function of HELLS in spontaneous OA. Thirty-two mice were randomly divided into 4 groups—control, miR-335-5p, miR-335-5p + HELLS, and miR-335-5p + HELLS-MUT—to study the effect of miR-335-5p on spontaneous OA. Each group contained 8 mice. The mice in each group were administered AAV through intra-articular injection every 8 weeks starting at 36 weeks of age.

The animals were provided with a standardized diet and housed in pathogen-free cages under controlled conditions of constant temperature and humidity. The circadian rhythm was maintained at a 12-h cycle. All experiments involving animals were approved and supervised by the Southern Medical University Animal Care and Use Committee (Guangzhou, China) (approval no. SMUL2021014).

### Statistical analysis

All results are presented as mean ± SD. Unpaired Student’s *t* tests were used to determine the statistical significance when comparing variances between 2 groups. One-way analysis of variance (ANOVA) and Tukey’s multiple comparison test or 2-way ANOVA and Sidak ’s multiple comparison test were used to evaluate the significance of differences across 3 or more groups. All statistical tests used were 2-sided. Statistical analyses and visualization were performed with GraphPad Prism 9.0 software (GraphPad Software Inc., La Jolla, CA, USA). Significance was attributed to *P* values below 0.05.

## Data Availability

The data are available from the corresponding authors on reasonable request. The raw RNA-sequencing files have been deposited in the Gene Expression Omnibus (GEO) database (GSE274759, GSE274760, and GSE274761).

## Supplementary Materials

Supplementary Methods Tables S1 to S3 Figs. S1 to S10 Reference [53]
