# Supplementary material for: MiR-335-5p Escaped from CircKIAA0586 Adsorption Contributes to Mechanical Overloading-Induced Cartilage Degeneration by Targeting Lymphoid-Specific Helicase
Source: Research (Wash D C). 2025 May 8;9:0694. doi: 10.34133/research.0694 (PMC12059312; doi:10.34133/research.0694)
Supplement: Supplementary file 1 — Supplementary Methods Tables S1 to S3 Figs. S1 to S10 Reference [53] [file research.0694.f1.docx]

**Supplementary Materials**

**MiR-335-5p Escaped from CircKIAA0586 Adsorption Contributes to Mechanical Overloading-induced Cartilage Degeneration by Targeting HELLS**

**(Short title: circKIAA0586 deficiency aggravates osteoarthritis)**

Haoyu Xie^1,2,3,4,†^, Yuheng Lu^5,†^, Jianying Pan^1,2,3,4,†^, Hua Zeng^1,2,3,4^, Zhicheng Zhang^1,2,3,4^, Jianbin Yin^1,2,3,4^, Jinjian Zhu^1,2,3,4^, Bingsheng Luo^1,2,3,4^, Dong Guo^1,2,3,4^, Chunyu Wu^1,2,3,4^, Chun Zeng^1,2,3,4^, Yan Shao^1,2,3,4^, Xiaochun Bai^6,*^, Daozhang Cai^1,2,3,4,*^, Haiyan Zhang^1,2,3,4,*^

^1^Department of Joint Surgery, Center for Orthopaedic Surgery, The Third Affiliated Hospital of Southern Medical University, Guangzhou, 510630, China.

^2^Department of Orthopedics, Orthopedic Hospital of Guangdong Province, Academy of Orthopedics·Guangdong Province, The Third Affiliated Hospital of Southern Medical University, Guangzhou, 510630, China.

^3^The Third School of Clinical Medicine, Southern Medical University, Guangzhou, 510630, China.

^4^Guangdong Provincial Key Laboratory of Bone and Joint Degeneration Diseases, Guangzhou, 510630, China.

^5^Department of Rehabilitation Medicine, Xijing Hospital, Fourth Military Medical University, Xi’an, 710032, China.

^6^State Key Laboratory of Organ Failure Research, Department of Cell Biology, School of Basic Medical Sciences, Southern Medical University, Guangzhou, 510515, China.

^†^These authors contributed equally to this work.

^*^Address correspondence to: zhhy0704@126.com (Haiyan Zhang); cdz@smu.edu.cn (Daozhang Cai); baixc15@smu.edu.cn (Xiaochun Bai)

**Supplementary Methods**

**Ethics statement**

The study has been carried out in accordance with The Code of Ethics of the World Medical Association (Declaration of Helsinki) for experiments involving humans. Before utilizing their clinical data for scientific investigation, all patients provided informed consent. This study was approved and supervised by the Ethics Committee of the Third Affiliated Hospital of Southern Medical University (Guangzhou, China). (Approval no. 2022-lunshen-053). Procedures were all performed in accordance with our institutional guidelines for animal research conforming to the Guide for the Care and Use of Laboratory Animals published by the US National Institutes of Health (NIH Publication No. 85-23, revised 1985). All experiments involving animals were approved and supervised by the Southern Medical University Animal Care and Use Committee (Guangzhou, China). (Approval no. SMUL2021014).

**Clinical cartilage sample collection**

During total knee arthroplasty, cartilage samples were collected from the tibial plateaus and femoral condyle of patients with OA (*n* = 10). The rough areas were classified as lesioned cartilage, whereas the smooth areas were classified as normal cartilage. Supplementary Table S1 provides a summary of the comprehensive patient information. Human cartilage samples were obtained from the Third Affiliated Hospital of Southern Medical University (Guangzhou, China).

**Animals**

From the Experimental Animal Center of the Southern Medical University (Guangzhou, China), 96 male C57BL/6J mice at the age of 8 weeks were obtained. Mice were raised until 16 weeks of age. Our previous research describes the method of destabilization of the medial meniscus model (DMM) surgery on the right knees of mice [52]. Briefly, the mice were anesthetized with 1% pentobarbital (50 mg·kg^−1^ body weight), and the medial meniscotibial ligament was incised after exposing the right knee joint capsule medially to the patellar tendon. After the assessment, the medial meniscus, right knee joint capsule, and skin were stitched. A simulated surgical procedure was performed on the right knee. The joint capsule was opened and closed without the intervention of the meniscus. For the investigation of the function of circKIAA0586 in experimental OA, 32 mice were randomly assigned to the following four groups: sham, control DMM, DMM + circKIAA0586, and DMM + circKIAA0586-MUT groups. For the assessment of the function of HELLS in experimental OA, 24 mice were randomly divided into three groups: sham, control DMM, and DMM + HELLS groups. Furthermore, 40 mice were divided into five groups, including sham, control DMM, DMM + miR-335-5p, DMM + miR-335-5p + HELLS, and DMM + miR-335-5p + HELLS-MUT groups, to investigate the function of miR-335-5p in OA. Each group contained eight mice. Geneseed (Guangzhou, China) constructed an adeno-associated virus (AAV) containing circKIAA0586 or circKIAA0586-MUT, which was packaged by Ubigene Biosciences Co., Ltd. (Guangzhou, China) along with a control AAV. AAVs containing HELLS or HELLS-MUT, as well as a control AAV, were produced and packaged by HanBio (Shanghai, China). GenePharma (Shanghai, China) supplied both the AAV that overexpressed miR-335-5p and the corresponding control AAV. The mice in each group received AAV intra-articular injection 2 weeks before and after DMM surgery. Mice in the control groups received control AAV for the same period. The right legs were collected after an 8-week postoperative period.

To create a spontaneous OA mouse model, 72 male C57BL/6J mice were obtained from the Experimental Animal Centre of Southern Medical University (Guangzhou, China) when they were 36 weeks old. Notably, mice were raised until 72 weeks of age. Three groups (control, circKIAA0586, and circKIAA0586-MUT groups) were formed by randomly dividing 24 mice to investigate the function of circKIAA0586 in spontaneous OA. Particularly, 16 mice were randomly divided into the control and HELLS groups to investigate the function of HELLS in spontaneous OA. Thirty-two mice were randomly divided into four groups: control, miR-335-5p, miR-335-5p + HELLS, and miR-335-5p + HELLS-MUT to study the effect of miR-335-5p on spontaneous OA. Each group contained eight mice. The mice in each group were administered AAV through intra-articular injection every 8 weeks starting at 36 weeks of age.

The animals were provided with a standardized diet and housed in pathogen-free cages under controlled conditions of constant temperature and humidity. The circadian rhythm was maintained at a 12-hour cycle.

**Histological analysis**

The knee joints were soaked in a solution of 4% paraformaldehyde for 24 hours for stabilization. Subsequently, they were decalcified in a solution of 0.5 mol·L^−1^ EDTA (pH 7.4) for at least 3 weeks, dehydrated, and then covered in paraffin. Sections of the samples, which were 4 meters thick, were stained with Safranin O/Fast Green.

The Osteoarthritis Research Society International scoring system was employed to grade cartilage degeneration levels [53]. Generally, sections were estimated with a score from 0 to 6, where 0 indicated intact cartilage, 0.5 indicated an intact surface with loss of proteoglycan, 1 indicated visible superficial fibrillation without loss of cartilage, 2 indicated vertical clefts and mild absence of surface lamina, 3 indicated vertical clefts or erosion to the calcified layer lesion affected < 25% of the articular surface, 4 indicated that the lesion reaches the calcified cartilage affecting 25%–50% of the articular surface, 5 indicated lesion spreads to the calcified cartilage affecting 50%–75% of the articular surface, 6 indicated lesion spreads to the calcified cartilage affecting ＞75% of the articular surface. The mean score obtained from two impartial and anonymous evaluators was used for subsequent statistical analyses.

**Isolation and culture of human primary chondrocyte**

Human femoral condyle cartilage samples were gathered and digested using 0.02% Collagenase P (COLLP-RO, Roche, Basel, Switzerland) for 16–18 hours at 37℃. After filtration of a 70 μm cell strainer, the chondrocytes were centrifuged for 5 minutes at 1,000 rpm. Following sedimentation, the cells were resuspended and cultured in Dulbecco’s Modified Eagle’s medium (DMEM; 10-013-CV, Corning, NY, USA) supplemented with 20% fetal bovine serum (35-081-CV, Corning). An incubator with a temperature of 37℃, 5% CO2, and 100% humidity was used to maintain the culture. Every 48 hours, the cell growth medium was changed after washing the cells with sterile polybutylene succinate (PBS) solution. When the cells in the culture dish covered over 80% of the bottom, they were treated with trypsin. Second-passage cells were generated by evenly dividing the treated cells between two fresh culture plates. Cells in the second passage were used for transfection and other procedures. The experiments were conducted within 3 days of sowing.

**Cyclic tensile strain loading of cells**

Human primary chondrocytes were subjected to cyclic tensile strain loading using previously published method [8]. The cells were seeded into silicon stretch chambers (BF-3001C, Flexcell, Burlington, NC, USA) at 50,000 cells per chamber density. After 48 hours of cultivation to guarantee chondrocyte attachment, a Flexcell-5000T mechanical stretch system (Flexcell) was employed to apply cyclic tensile strain (0.5 Hz, 20% intensity) to the cells for 24 hours in a CO_2_ incubator. The chambers were kept constant for the control cells and cultured without mechanical loading.

**Cell transfection**

Chondrocytes were transfected with circKIAA0586, circKIAA0586-MUT, HELLS, HELLS-MUT overexpression plasmid, or the corresponding control plasmid using Lipofectamine 3000 (L3000015, Thermo Fisher Scientific, Waltham, MA, USA) following the manufacturer’s recommendations. Plasmids overexpressing circKIAA0586, circKIAA0586-MUT, and the control plasmid were obtained from Geneseed (Guangzhou, China). Plasmids overexpressing HELLS, HELLS-MUT, and control plasmids were constructed using Tsingke Biotechnology (Beijing, China). MiR-335-5p mimic, miR-negative control (NC), miR-335-5p inhibitor, inhibitor- NC, HELLS-specific siRNA, and control siRNA (Tsingke) were transfected into chondrocytes using Lipofectamine RNAiMAX (13778075, Thermo Fisher). For the specific inhibition of circKIAA0586, CRISPR/Cas9 knockdown plasmids targeting circKIAA0586 cyclization elements were constructed by HanBio (Shanghai, China) and transfected into chondrocytes using Lipofectamine 3000 (Thermo Fisher Scientific). All transfection experiments were performed using chondrocytes at 70% confluence.

**CCK-8 assay**

The viability of chondrocytes was determined by performing the Cell Counting Kit-8 (CCK-8) assay (KGA9310, KeyGEN biotech, Nanjing, China). Primary human chondrocytes were cultured on 96-well plates at a density of 3,000 cells per well. Once the cells had reached around 50% confluence, they were exposed to 20% cyclic tensile strain (CTS) loading at 0.5 Hz for 0, 6, 12 and 24 hours, with five replicates for each group. And adding 10 μl of CCK8 solution to each well and incubating for 2 hours. Optical density was assessed at a wavelength of 450 nm. All operation procedures were conducted following the manufacturer’s instructions.

**Senescence-associated β-galactosidase staining and toluidine blue staining**

The activity of galactosidase (SA-βGal) was determined using a staining kit (G1580, Solarbio, Beijing, China) following the manufacturer’s guidelines. Chondrocytes were placed in either 6-well plates or silicon stretch chambers, then treated with a fixed buffer for 10 minutes, rinsed, and exposed to a staining solution for 16 hours at 37℃. Toluidine blue staining was performed using a toluidine blue staining solution (G2543, Solarbio). Chondrocytes were seeded onto 6-well plates or silicon stretch chambers, fixed at room temperature for 10 min, rinsed, and exposed to the staining solution for 10 min at room temperature, followed by five PBS washes (5 min each). Five visual fields were randomly selected from each group for cell counts. The number of positive cells and the total number of cells were counted by Image J. The positive cell rate was obtained by dividing positive cells number by total cells number and then multiplying by 100%.

**Quantitative RT-PCR**

Total RNA was extracted from human primary chondrocytes and human cartilage tissues using AG21102 RNAex Pro reagent (Accurate Biotechnology, Changsha, China). To quantify mRNA and circRNA, total RNA (1 mg) was treated with a genomic DNA remover and subsequently reverse transcribed using the Evo M-MLV RT kit (AG11728, Accurate Biotechnology). In each PCR reaction, 10 µL of 2× SYBR Green Pro Taq HS Premix (AG11701, Accurate Biotechnology), 10 µM of forward and reverse primers, and 100 ng of complementary DNA (cDNA) were utilized. For miRNA quantification, 1 mg of total RNA was purified, followed by reverse transcription using specific stem-loop primers and the miRNA 1st Strand cDNA Synthesis Kit (MR101-01; Vazyme, Nanjing, China). The 2× miRNA Universal SYBR qPCR Master Mix, corresponding primers, and mQ primer R (MQ101-01, Vazyme) were included in the DNA template. Each assay was conducted thrice and standardized to either the mRNA housekeeping gene or the miRNA housekeeping gene RNU6-1 (U6). Supplementary Table S2 presents a comprehensive list of all the primer details.

**RNase R digestion assay**

Total RNAs were reacted with or without 3 U/μg of RNase R (R0301, Geneseed) at 37℃ for 10 min, and the resulting RNA subsequently underwent qRT-PCR detection.

**RNA sequencing**

Total RNA and miRNA were extracted from human primary chondrocytes suffered from mechanical overloading and control cells by usage of the miRNeasy® Mini Kit (217004, Qiagen, Dusseldorf, Germany). CircRNA sequencing was performed at Geneseed Biotech Co., Ltd. (Guangzhou, China). MiRNA and mRNA sequencing was performed at BGI Genomics (Shenzhen, China). Differentially expressed RNAs (Supplementary Table S4-S6) between two groups of human primary chondrocytes suffered from mechanical overloading and control cells were carried out with a cut-off false discovery rate < 0.05 and antibody (log2 fold change) > 1. The R programming language was used to generate heatmaps, Venn diagrams, and scatterplots. The raw RNA-sequencing files have been deposited in the Gene Expression Omnibus (GEO) database (www.ncbi.nlm.nih.gov/geo) (GSE274759, GSE274760, and GSE274761).

**Western blotting**

To lyse cells grown in 6-well dishes, 100 μL of RIPA buffer (FD009, Fudebio, Hangzhou, China) with added protease inhibitors (FD1001, Fudebio) and phosphatase inhibitors (FD1002, Fudebio) was utilized. The total protein concentration using a bicinchoninic acid protein quantitative kit (FD2001, Fudebio) was used according to the manufacturer’s instructions. Proteins were subjected to SDS-PAGE and subsequently transferred to an Immobilon-PSQ PVDF Membrane (ISEQ00010; Millipore, Billerica, MA, USA). Following blocking at room temperature for 1 hour with 5% skim milk in 50 mmol·L^−1^ Tris-buffered saline (TBS) (pH 7.4) containing 0.1% Tween-20 (TBST), the membranes were then exposed to primary antibodies diluted in 5% BSA dissolved in TBST and incubated overnight at 4℃. After five rounds of washing with TBST for 5 min each, the membranes were incubated with secondary antibodies (diluted 1:5,000 in TBST) at room temperature for 1 h. Immunoblotting signals were detected with FDbio-Dura ECL (FD8020; Fudebio) and visualized with Tanon 5200 Imaging System (Tanon, Shanghai, China). The antibodies used for western blotting were shown in Supplementary Table S3. All of the full-length original western blots are provided as supplementary material.

**Co-immunoprecipitation (Co-IP) assay**

A classic IP/Co-IP kit (IK-1004, Biolinkedin, Shanghai, China) was used to conduct co-IP assays following the guidelines provided by the manufacturer. Controls and chondrocytes that underwent cyclic tensile strain loading were lysed in IP lysis buffer supplemented with a protease inhibitor cocktail for 20 min on ice. The supernatants were collected after centrifuging lysates at 16,000 g for 10 min. Supernatants containing proteins were subsequently incubated with 2μg rabbit anti-CDCA7 antibody (15249-1-AP, Proteintech) or 2 μg rabbit IgG as control in a rotation machine at 4℃ overnight. Afterward, the protein lysates were mixed with 25 μL of Protein A/G Magnetic Beads (Biolinkedin) and gently rotated for 2 hours at room temperature. The immunoprecipitates were detected using immunoblotting.

**Dual-luciferase reporter assay**

Luciferase-reporting plasmids, including circKIAA0586, HELLS, and their mutant versions, were constructed by HanBio (Shanghai, China). SW1353 cells were transfected with a luciferase reporter plasmid and miR-335-5p mimic/miR-NC (Tsingke) using Lipofectamine 3000 (Thermo Fisher Scientific) following the manufacturer’s guidelines after being cultured in 12-well plates until they reached approximately 70% confluence. Luciferase activity was determined using a dual-luciferase reporter assay system (E1910, Promega, Madison, WI, USA) 48 hours after co-transfection. For subsequent statistical analyses, firefly luciferase activity was normalized to that of Renilla luciferase.

**RNA immunoprecipitation (RIP) assay**

RIP experiments were conducted using the EZ-Magna RIP RNA-Binding Protein Immunoprecipitation Kit (17-701, Millipore). MiR-NC and miR-335-5p mimics were transfected into SW1353 cells. Approximately ten million cells were subsequently moved to a pellet of equal volume and dissolved using 100 μL of RIP Lysis Buffer, which included protease and RNase inhibitors. Afterward, the cell lysates were rotated overnight at 4℃ with either an antibody targeting Ago2 (03-110, Sigma) or mouse IgG in a rotation machine. After treatment with proteinase K buffer, immunoprecipitated RNA was extracted and purified using reverse transcription (Accurate Biotechnology) and the RNeasy MinElute Cleanup Kit (74204, Qiagen). circRNA expression was determined using quantitative PCR.

**RNA pulldown assay**

A miRNA pulldown kit (Bes5108, BersinBio, Guangzhou, China) was used to conduct RNA pulldown assays following the manufacturer’s guidelines. Biotinylated miR-335-5p or control probes (GenePharma) were transfected into SW1353 cells. Around ten million SW1353 cells were collected and lysed in a lysis solution containing protease inhibitors, RNase Inhibitors, and Dithiothreitol for 20 min on ice. After centrifuging at 13,000 g for 5 minutes, the lysates were discarded, and the supernatants were gently rotated with 40 μL of Streptavidin magnetic beads for 4 hours at 4℃. RNA bound to the probes was eluted and extracted for qRT-PCR analysis.

**Immunohistochemistry (IHC) and immunofluorescence (IF) analyses**

Following the removal of paraffin and restoration of hydration, the slides were rinsed thrice in PBS for 5 min each. The antigen was retrieved by immersing the slides in a citric acid buffer (10 mmol·L^−1^ citric acid, pH 6.0), heating in a water bath at 95℃ for 15 minutes, and then washing thrice with PBS. The slides were subsequently treated with 3% hydrogen peroxide for 10 min to prevent endogenous peroxidase activity, followed by three additional washes with PBS. Subsequently, the slides were soaked in goat serum with a concentration of 10% for 1 hour at room temperature and then subjected to primary antibodies at 4℃ overnight. IF or IHC secondary antibodies were then incubated at room temperature for 1 hour. The IHC slides were treated with hematoxylin and 3,3’-diaminobenzidine (DAB, ZSGB-Bio, Beijing, China), dehydrated, and mounted. The IF slides were mounted after staining with 4,6-diamidino-2-phenylindole (DAPI) solution (Thermo Fisher Scientific). Digital images were captured using an BX53 microscope (Olympus, Tokyo, Japan). The number of positive cells and the total number of cells were counted by Image J. The positive cell rate was obtained by dividing positive cells number by total cells number and then multiplying by 100%. All chondrocytes from the surface of cartilage to the boundaries between cartilage and subchondral bone were included in the count. The antibodies used for IHC/IF were shown in Supplementary Table S3.

**Immunocytochemistry**

Human primary chondrocytes were immobilized by treating the slides with 4% paraformaldehyde for 10 min, followed by washing with PBS thrice. Subsequently, the slides were treated with 0.5% Triton X-100 (P0096, Beyotime) for 15 min to permeabilize the cell membrane, followed by washing thrice with PBS. Afterward, the slides were blocked with 10% goat serum for 30 minutes at room temperature. Subsequently, the slides were subjected to suitable primary antibodies overnight at 4℃ and then treated with secondary antibodies labeled with Alexa-488 or Alexa-594 that matched the species (1:200, Invitrogen) at room temperature for 1 hour. The immunocytochemistry slides were stained with a DAPI staining solution purchased from Thermo Fisher Scientific and then mounted. Digital images were captured using an BX53 microscope (Olympus). The antibodies used for immunocytochemical staining were shown in Supplementary Table S3.

**RNA fluorescent in situ hybridization (FISH)**

Slides containing crawling human primary chondrocytes or mouse joint sections were used for the FISH assay. GenePharma (Shanghai, China) created miR-335-5p probes labeled with CY3 and circKIAA0586 probes labeled with FAM. Fluorescent RNA in situ hybridization kits, F12201 and F22201 (GenePharma), were used to verify the probe signals on cell-crawling slides and paraffin sections, respectively, following the manufacturer’s instructions.

**Bioinformatics analysis**

The circRNA upstream of miR-335-5p was predicted using StarBase (starbase.sysu.edu.cn). The mRNA targets of miR-335-5p were predicted using TargetScan (www.targetscan.org) and miRanda (www.microrna.org/microrna).

**Statistical analysis**

All results are presented as mean ± SD. Unpaired Student’s *t*-tests were used to determine the statistical significance when comparing variances between two groups. One-way analysis of variance (ANOVA) and Tukey’s multiple comparison test or two-way ANOVA and Sidak's multiple comparison test were used to evaluate the statistical significance of differences across three or more groups. All statistical tests used were two-sided. Statistical analyses and visualization were performed using GraphPad Prism 9.0 software (GraphPad Software Inc., La Jolla, CA, USA). Significance was attributed to *P* values below 0.05.

**Supplementary Tables**

**Supplementary Table S1. Clinical characteristics of recruited OA patients (n = 10).**

| **Characteristics** | **N Mean ± SD** | **Range or Percentage** |
| --- | --- | --- |
| Age | 66.40 ± 4.38 | 62-75 |
| Female | 5 | 50% |
| Male | 5 | 50% |
| KL grade | 3.80 ± 0.42 | 3-4 |
| Height (m) | 1.65 ± 0.10 | 1.49-1.83 |
| Weight (kg) | 69.40 ± 9.88 | 55.5-82 |
| BMI (kg/m^2^) | 25.39 ± 2.04 | 27.9-21.4 |

**Supplementary Table S2. Primers and sequences used in this study.**

| **Gene** | **Type** | **Sequences** |
| --- | --- | --- |
| COL2A1 | Forward primer | CCAGATGACCTTCCTACGCC |
|  | Reverse primer | TTCAGGGCAGTGTACGTGAAC |
| ACAN | Forward primer | GTGCCTATCAGGACAAGGTCT |
|  | Reverse primer | GATGCCTTTCACCACGACTTC |
| MMP3 | Forward primer | CTGGACTCCGACACTCTGGA |
|  | Reverse primer | CAGGAAAGGTTCTGAAGTGACC |
| MMP13 | Forward primer | CCAGACTTCACGATGGCATTG |
|  | Reverse primer | GGCATCTCCTCCATAATTTGGC |
| ADAMTS5 | Forward primer | GGGCACTGGCTACTATGTGG |
|  | Reverse primer | CGTCACAGCCAGTTCTCACA |
| p16 | Forward primer | GGGTTTTCGTGGTTCACATCC |
|  | Reverse primer | CTAGACGCTGGCTCCTCAGTA |
| P53 | Forward primer | GTTGATTCCACACCCCCGC |
|  | Reverse primer | CGCCTCACAACCTCCGTCAT |
| HELLS | Forward primer | ACACTTCCTAACTGGATGGCT |
|  | Reverse primer | ACTCCCTGATTAGACGGCAC |
| PRKAA2 | Forward primer | CTCGCCTCTAGTCCTCCATC |
|  | Reverse primer | CGGTTTGCTCTGACTTCGG |
| CD55 | Forward primer | CCAAGGTCCCACCAACAG |
|  | Reverse primer | ACTAGCGTCCCAAGCAAAC |
| CRIM1 | Forward primer | CCTCACGCACCCAGGATTC |
|  | Reverse primer | GTACGCTGTTATTGCGGGAC |
| ATP1B1 | Forward primer | GTATAACCCAAATGTCCTTCCCG |
|  | Reverse primer | CACTGTACCCAATGTTCTCACC |
| ACTB | Forward primer | AGAGCTACGAGCTGCCTGAC |
|  | Reverse primer | AGCACTGTGTTGGCGTACAG |
| KIAA0586 | Forward primer | GGAAGGAACAAGCAGTGGCG |
|  | Reverse primer | TGTCACCCAGCATGACAGCA |
| hsa_circ_0000698 | Forward primer | GCCGATAAGGTCCAGCAGTT |
|  | Reverse primer | GGTCACCACCGCATGTTTT |
| hsa_circ_0001006 | Forward primer | GTGAACTGCACGATAAAGGAAC |
|  | Reverse primer | GAATAGGCTGGCACCAAACA |
| hsa_circ_0004791 | Forward primer | GGCAGCACAGAGCTACATTCG |
|  | Reverse primer | CCTTCTGGTCACCACCGCAT |
| circKIAA0586  (has_circ_0102233) | Forward primer | TTGCTCACCTTCATCACCTGCTA |
|  | Reverse primer | CGCCACTGCTTGTTCCTTC |
| circKIAA0586  (convergent) | Forward primer | CCACCTGCTGGAGTGATTG |
|  | Reverse primer | TCGAGATGATGGAGGAATAGAAG |
| GAPDH  (convergent) | Forward primer | CAAGGCTGTGGGCAAGGTC |
|  | Reverse primer | GTCGCTGTTGAAGTCAGAGGAGA |
| GAPDH  (divergent) | Forward primer | GGAGTCCACTGGCGTCTTCA |
|  | Reverse primer | GGCTGCCCATTCATTTCCTT |
| miR-335-5p | Forward primer | CGCGTCAAGAGCAATAACGAA |
|  | Stem loop primer | GTCGTATCCAGTGCAGGGTCCGAGGTATTCGCACTGGATACGACACATTT |
| miR-337-3p | Forward primer | CGCGCTCCTATATGATGCCT |
|  | Stem loop primer | GTCGTATCCAGTGCAGGGTCCGAGGTATTCGCACTGGATACGACGAAGAA |
| miR-3613-5p | Forward primer | GCGCGTGTTGTACTTTTTTTT |
|  | Stem loop primer | GTCGTATCCAGTGCAGGGTCCGAGGTATTCGCACTGGATACGACGAACAA |
| miR-149-5p | Forward primer | CGTCTGGCTCCGTGTCTTC |
|  | Stem loop primer | GTCGTATCCAGTGCAGGGTCCGAGGTATTCGCACTGGATACGACGGGAGT |
| miR-369-5p | Forward primer | CGCGAGATCGACCGTGTTAT |
|  | Stem loop primer | GTCGTATCCAGTGCAGGGTCCGAGGTATTCGCACTGGATACGACGCGAAT |
| miR-496 | Forward primer | CGCGTGAGTATTACATGGCC |
|  | Stem loop primer | GTCGTATCCAGTGCAGGGTCCGAGGTATTCGCACTGGATACGACGAGATT |
| miR-199b-3p | Forward primer | GCGCGACAGTAGTCTGCACAT |
|  | Stem loop primer | GTCGTATCCAGTGCAGGGTCCGAGGTATTCGCACTGGATACGACTAACCA |
| miR-199a-5p | Forward primer | CGCGCCCAGTGTTCAGACTAC |
|  | Stem loop primer | GTCGTATCCAGTGCAGGGTCCGAGGTATTCGCACTGGATACGACGAACAG |
| miR-221-3p | Forward primer | CGCGAGCTACATTGTCTGCTG |
|  | Stem loop primer | GTCGTATCCAGTGCAGGGTCCGAGGTATTCGCACTGGATACGACGAAACC |
| miR-199b-5p | Forward primer | CGCGCCCAGTGTTTAGACTAT |
|  | Stem loop primer | GTCGTATCCAGTGCAGGGTCCGAGGTATTCGCACTGGATACGACGAACAG |
| RNU6-1 (U6) | Forward primer | CTCGCTTCGGCAGCACA |
|  | Stem loop primer | AACGCTTCACGAATTTGCGT |
| Cy3-miR-335-5p | Probes for FISH | ACATTTTTCGTTATTGCTCTTGA |
| Fam-circKIAA0586 | Probes for FISH | GCCACTGCTTGTTCCTTCCACTTTTTCAGCACATATTTCT |
| Bio-miR-335-5p | Probes for RNA pull-down | UCAAGAGCAAUAACGAAAAAUGU |
| Bio-control probe | Probes for RNA pull-down | AAAAAAAAAAAAAAAAAAAAAA |
| miR-335-5p mimics | minicssense | UCAAGAGCAAUAACGAAAAAUGU |
|  | minicsantisense | ACAUUUUUCGUUAUUGCUCUUGA |
| mimics control | minicssense | UCACAACCUCCUAGAAAGAGUAGA |
|  | minicsantisense | UCUACUCUUUCUAGGAGGUUGUGA |
| miR-335-5p inhibitor | inhibitor | ACAUUUUUCGUUAUUGCUCUUGA |
| Inhibitor control | inhibitor | UCUACUCUUUCUAGGAGGUUGUGA |
| HELLS siRNA-1 | siRNA-forward | GAAGUGAAUAUCCCUGUAGAA |
|  | siRNA-Reverse | UUCUACAGGGAUAUUCACUUC |
| HELLS siRNA-2 | siRNA-forward | GCCAUUGUGAACCGUACAATT |
|  | siRNA-Reverse | UUGUACGGUUCACAAUGGCTT |
| siRNA control | siRNA-forward | UUCUCCGAACGUGUCACGUTT |
|  | siRNA-Reverse | ACGUGACACGUUCGGAGAATT |
| circKIAA0586 sgRNA-1 | cas9-gRNA | CAGGCACGCGTCACCATGCC |
| circKIAA0586 sgRNA-2 | cas9-gRNA | GTAGAAGAATTGCTTGAACC |

**Supplementary Table S3.** **Antibodies used in this study.**

| **Antigen** | **Source and Catalog** | **Host species** | **Application** | **Dilution** |
| --- | --- | --- | --- | --- |
| collagen Ⅱ | Sigma-Aldrich, SAB4500366 | Rabbit | WB | 1:1,000 |
| MMP13 | Proteintech, 18165-1-AP | Rabbit | WB | 1:1,000 |
| MMP3 | ABclonal, A11418 | Rabbit | WB | 1:1,000 |
| Aggrecan | ABclonal, A11691 | Rabbit | WB | 1:1,000 |
| ADAMTS5 | Abcam, ab41037 | Rabbit | WB | 1:1,000 |
| p16 | CST, 18769s | Rabbit | WB | 1:1,000 |
| p53 | Abcam, ab1101 | Mouse | WB | 1:1,000 |
| γ-H2A.X | CST, 9718s | Rabbit | WB | 1:1,000 |
| HELLS | Proteintech, 11955-1-AP | Rabbit | WB | 1:1,000 |
| CDCA7 | Proteintech, 15249-1-AP | Rabbit | WB | 1:1,000 |
| GAPDH | Fudebio, FD0063 | Mouse | WB | 1:5,000 |
| Goat Anti-Rabbit IgG (H+L) | Jackson ImmunoResearch Laboratories, 111-035-003 | Goat | WB | 1:5,000 |
| Goat Anti-Mouse IgG (H+L) | Jackson ImmunoResearch Laboratories, 115-035-003 | Goat | WB | 1:5,000 |
| HELLS | Proteintech, 11955-1-AP | Rabbit | IHC | 1:100 |
| Goat Anti-Rabbit IgG (H+L) | Jackson ImmunoResearch Laboratories, 111-035-003 | Goat | IHC | 1:200 |
| collagen Ⅱ | Sigma-Aldrich, SAB4500366 | Rabbit | IF | 1:100 |
| MMP13 | Proteintech, 18165-1-AP | Rabbit | IF | 1:100 |
| p16 | CST, 29271s | Rabbit | IF | 1:50 |
| γ-H2A.X | CST, 9718s | Rabbit | IF | 1:100 |
| Anti-Rabbit IgG (H+L), Alexa Fluor™ 488 | Invitrogen, A-11034 | Goat | IF | 1:400 |
| Anti-Rabbit IgG (H+L), Alexa Fluor™ 594 | Invitrogen, A-21207 | Donkey | IF | 1:400 |
| CDCA7 | Proteintech, 15249-1-AP | Rabbit | ICC | 1:100 |
| HELLS | Santa Cruz, sc-46665 | Mouse | ICC | 1:50 |
| Anti-Rabbit IgG (H+L), Alexa Fluor™ 488 | Invitrogen, A-11034 | Goat | ICC | 1:500 |
| Anti-Mouse IgG (H+L), Alexa Fluor™ 594 | Invitrogen, A-21203 | Donkey | ICC | 1:500 |
| CDCA7 | Proteintech, 15249-1-AP | Rabbit | IP | 1:100 |
| Rabbit IgG control | Proteintech, 30000-0-AP | Rabbit | IP | 1:100 |
| Ago2 | Sigma-Aldrich, 03-110 | Mouse | RIP | 1:100 |
| Mouse IgG control | Sigma-Aldrich, 03-119 | Mouse | RIP | 1:100 |

**Supplementary Figures**


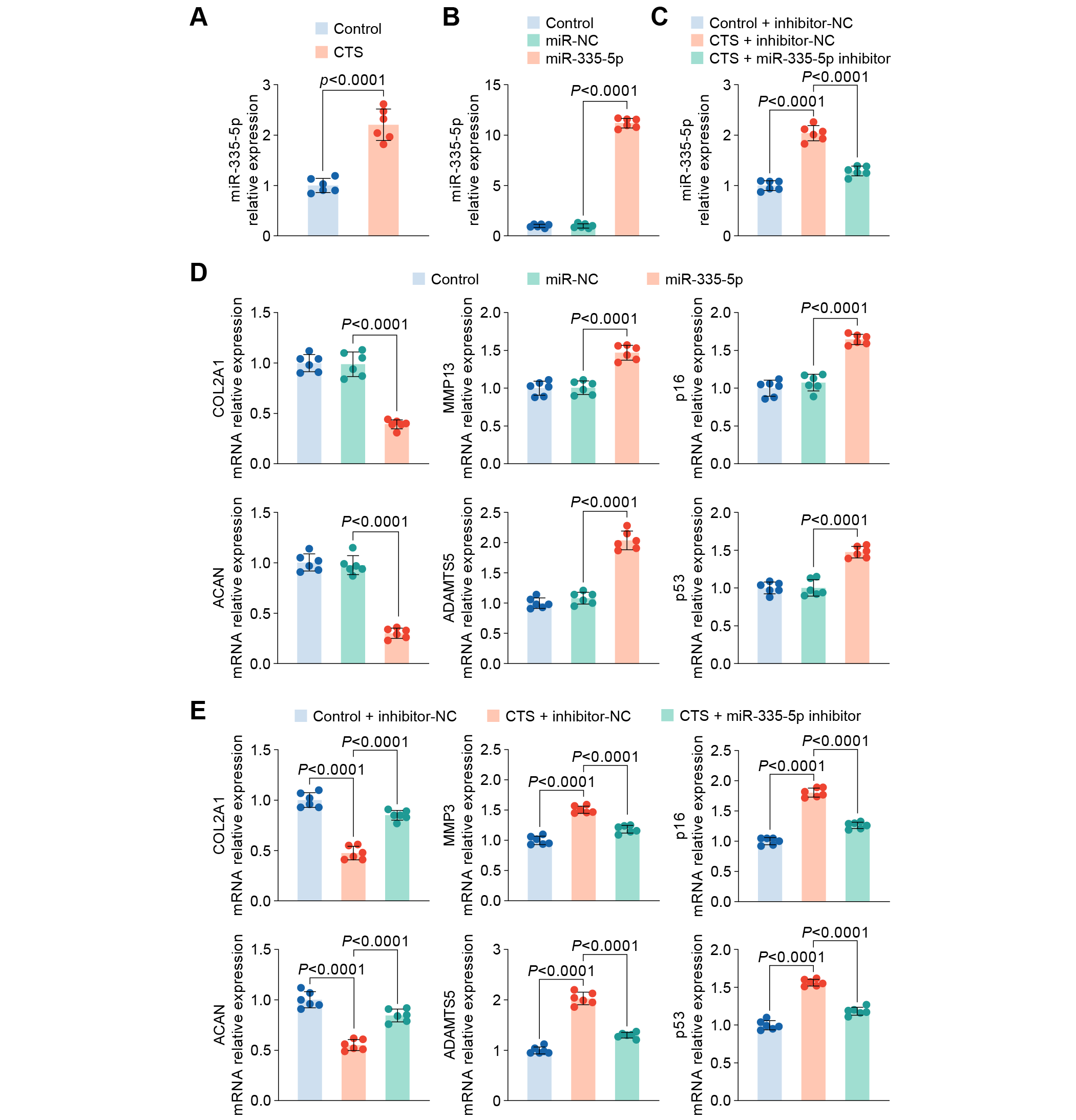


**Figure. S1. MiR-335-5p mediates mechanical overloading-induced chondrocyte senescence *in vitro*.**

**(A)** Quantitative reverse transcription polymerase chain reaction (qRT-PCR) analysis detected miR-335-5p levels in human primary chondrocytes treated with 20% cyclic tensile strain (CTS) loading for 24 hours and control cells (n = 6 per group).

**(B)** qRT-PCR analysis of miR-335-5p in controls and human primary chondrocytes overexpressing miR-335-5p or miR-NC (*n* = 6 per group).

**(C)** qRT-PCR analysis of miR-335-5p in controls and human primary chondrocytes with or without miR-335-5p inhibition after treated with 20% CTS loading for 24 hours (*n* = 6 per group).

**(D)** qRT-PCR analysis of COL2A1, MMP13, ACAN, ADAMTS5 and senescence markers (p16 and p53) in controls and human primary chondrocytes overexpressing miR-335-5p or miR-NC (*n* = 6 per group).

**(E)** qRT-PCR analysis of COL2A1, MMP3, ACAN, ADAMTS5, and senescence markers (p16 and p53) in controls and human primary chondrocytes with or without miR-335-5p inhibition after treated with 20% CTS loading for 24 hours (*n* = 6 per group).

The statistics are presented as the mean ± standard deviation.


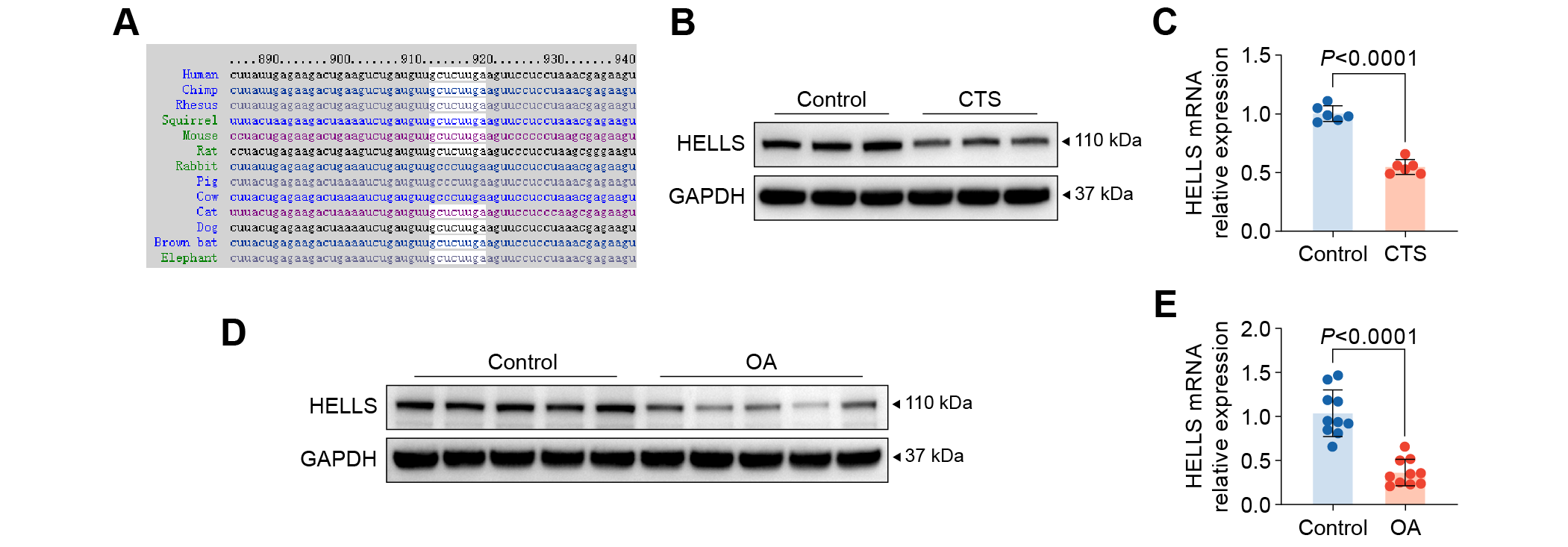


**Figure. S2. The expression of HELLS in osteoarthritis patients and OA chondrocytes.**

**(A)** Schematic illustration to show the conserved CDS-binding site of HELLS mRNA to miR-335-5p, as predicted using TargetScan.

**(B, C)** Immunoblotting (B) and quantitative reverse transcription polymerase chain reaction (qRT-PCR) (C) analysis of HELLS protein and mRNA levels in human primary chondrocytes treated with 20% cyclic tensile strain (CTS) loading for 24 hours and control cells (*n* = 6 per group).

**(D, E)** Immunoblotting (D) and qRT-PCR (E) analysis of HELLS protein and mRNA levels in knee cartilage of controls and OA patients (*n* = 10 samples per group).

Data are presented as the mean ± standard deviation.


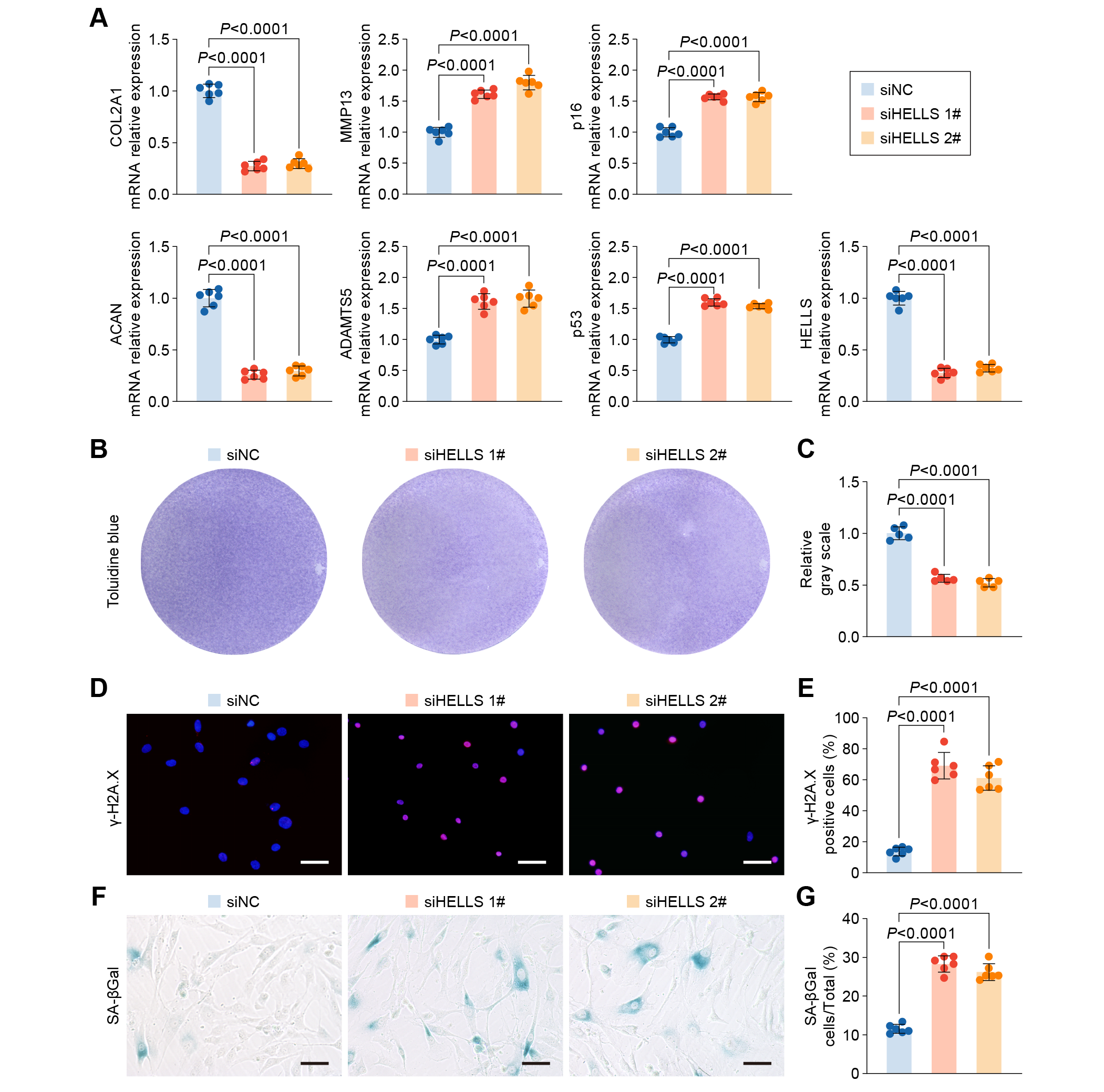


**Figure. S3. Knockdown of HELLS accelerates chondrocyte senescence *in vitro*.**

**(A)** Quantitative reverse transcription polymerase chain reaction (qRT-PCR) analysis of HELLS, COL2A1, MMP13, ACAN, ADAMTS5 and senescence markers (p16 and p53) in human primary chondrocytes transfected with siHELLS or siNC (*n* = 6 per group).

**(B, C)** Representative images (B) and quantification (C) of toluidine blue staining of human primary chondrocytes transfected with siHELLS or siNC.

**(D, E)** Representative images (D) and quantification (E) of γ-H2A.X immunofluorescence staining in human primary chondrocytes transfected with siHELLS or siNC (*n* = 6 per group). Scale bars: 40 μm.

**(F, G)** Representative images (F) and quantification (G) of SA-βGal staining in human primary chondrocytes transfected with siHELLS or siNC (*n* = 6 per group). Scale bars: 20 μm. The statistics are shown as the mean ± standard deviation.


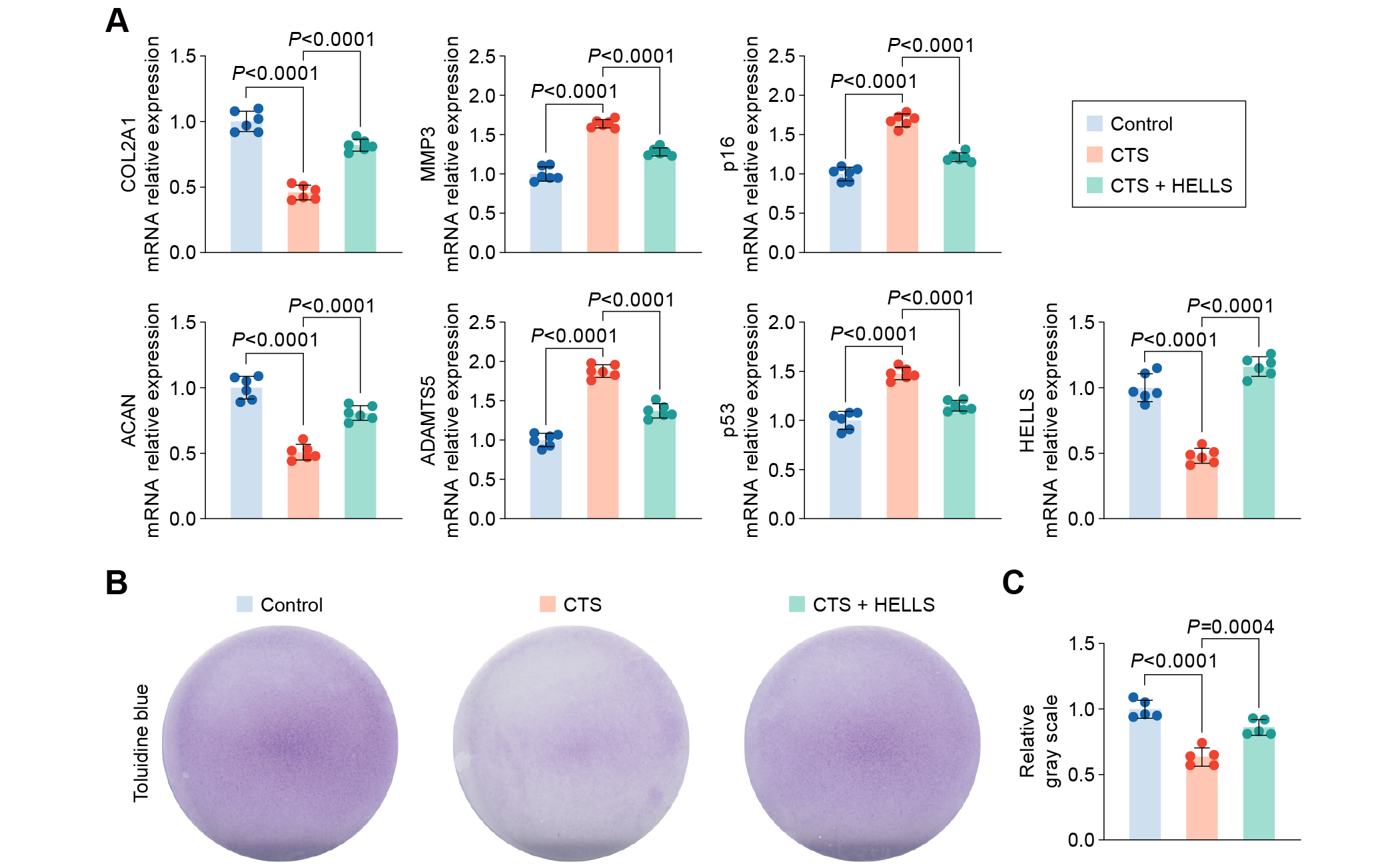


**Figure. S4. HELLS alleviates mechanical overloading-induced chondrocyte senescence *in vitro*.**

**(A)** Quantitative reverse transcription polymerase chain reaction (qRT-PCR) analysis of HELLS, COL2A1, MMP3, ACAN, ADAMTS5 and senescence markers (p16 and p53) in controls and human primary chondrocytes with or without HELLS overexpression after treatment with 20% cyclic tensile strain (CTS) loading for 24 hours (*n* = 6 per group).

**(B, C)** Representative images (B) and quantification (C) of toluidine blue staining of controls and human primary chondrocytes with or without HELLS overexpression after treatment with 20% CTS loading lasting 24 hours.

The statistics are shown as the mean ± standard deviation.


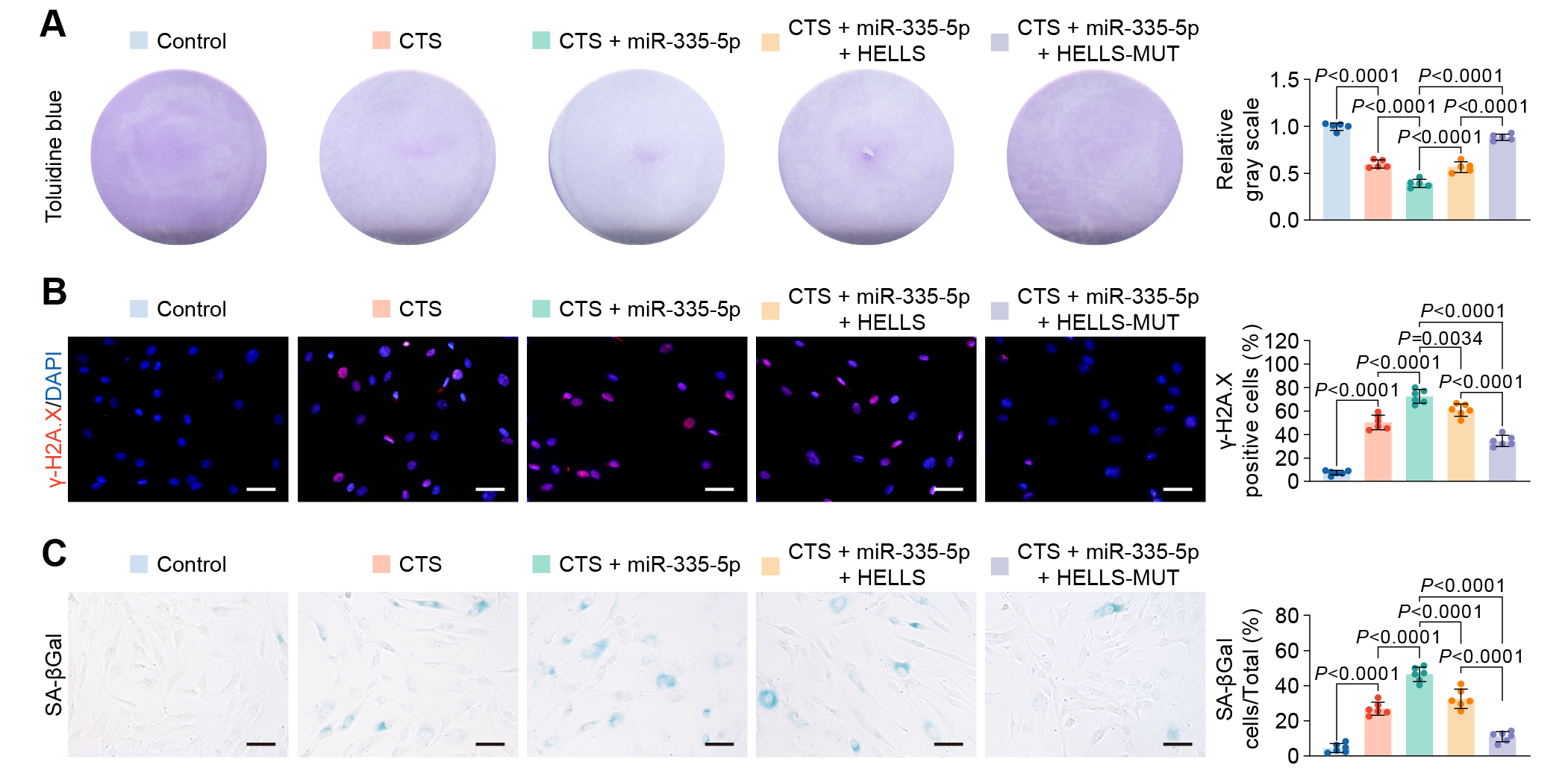


**Figure. S5. HELLS with synonymous mutation for miR-335-5p presents protection against osteoarthritis *in vitro*.**

**(A)** Representative images and quantification of toluidine blue staining of human primary chondrocytes administered with 20% cyclic tensile strain (CTS) loading for 24 hours after co-transfection with miR-335-5p and HELLS or HELLS-MUT plasmids.

**(B, C)** Representative images and quantification of γ-H2A.X immunofluorescence staining (B) and SA-βGal staining (C) in human primary chondrocytes administered with 20% CTS loading for 24 hours after co-transfection with miR-335-5p and HELLS or HELLS-MUT plasmids (n = 6 per group). Scale bars: 40 μm (B) and 20 μm (C).

The statistics are presented as the mean ± standard deviation.


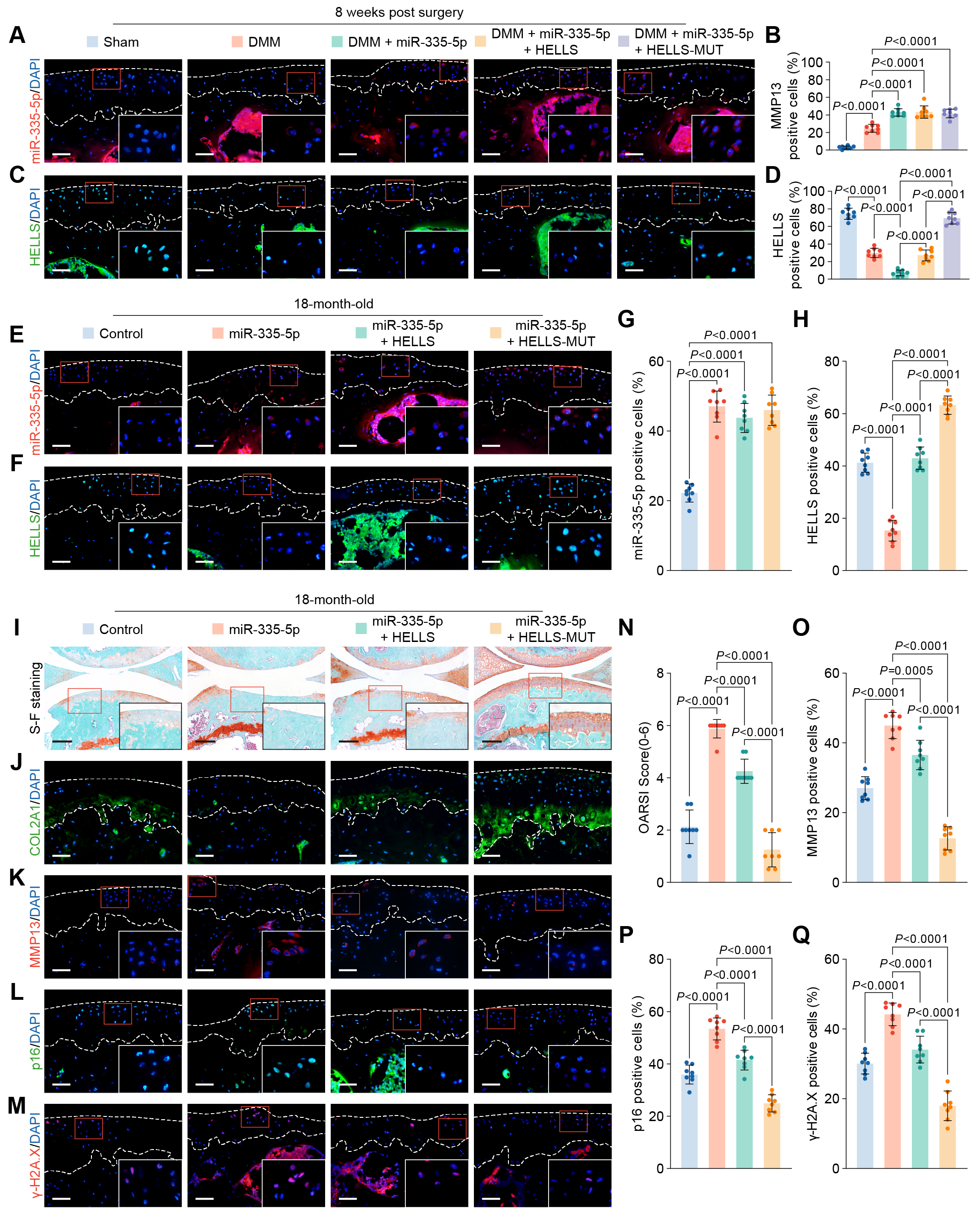


**Figure. S6. HELLS with synonymous mutation for miR-335-5p presents protection against osteoarthritis *in vivo*.**

**(A**-**D)** Representative images and quantification of miR-335-5p FISH staining (A, B) and HELLS immunofluorescence staining (C, D) in knee cartilage from sham mice and destabilization of the medial meniscus (DMM) mice administered miR-335-5p AAV alone or with HELLS AAV or HELLS-MUT AAV at 8 weeks after DMM surgery (*n* = 8 per group). Scale bar: 40 μm.

**(E**-**H)** Representative images and quantification of miR-335-5p FISH staining (E, G) and HELLS immunofluorescence staining (F, H) in knee cartilage from 18-month-old aged spontaneous OA mice administered miR-335-5p AAV alone or with HELLS AAV or HELLS-MUT AAV (*n* = 8 per group). Scale bar: 40 μm.

**(I**-**Q)** Representative images and quantification of safranin O and fast green staining (I, N) and immunofluorescence staining of COL2A1 (J), MMP13 (K, O), p16 (L, P), and γ-H2A.X (M, Q) in knee cartilage from 18-month-old aged spontaneous OA mice administered miR-335-5p AAV alone or with HELLS AAV or HELLS-MUT AAV (*n* = 8 per group). Scale bars: 100 μm (first row) and 40 μm (other rows).

All chondrocytes from the surface of cartilage (white dotted line on top) to the boundaries between cartilage and subchondral bone (white dotted line below) were included in the count. The statistics are presented as the mean ± standard deviation.


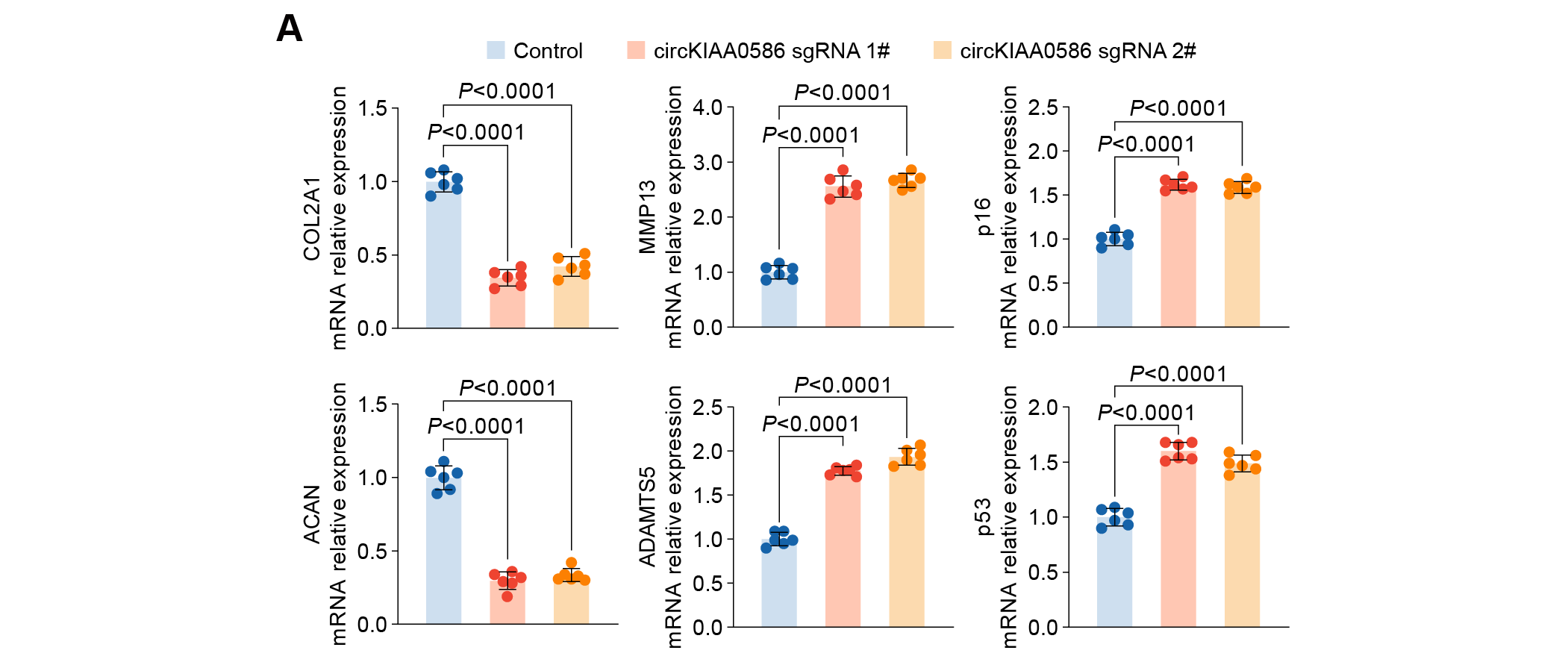


**Figure. S7. CircKIAA0586 regulates chondrocyte senescence and metabolism *in vitro*.**

**(A)** Quantitative reverse transcription polymerase chain reaction (qRT-PCR) analysis of COL2A1, MMP13, ACAN, ADAMTS5 and senescence markers (p16, p53) in human primary chondrocytes transfected with circKIAA0586 knockdown plasmids and control cells (*n* = 6 per group).

Data are presented as the mean ± standard deviation.


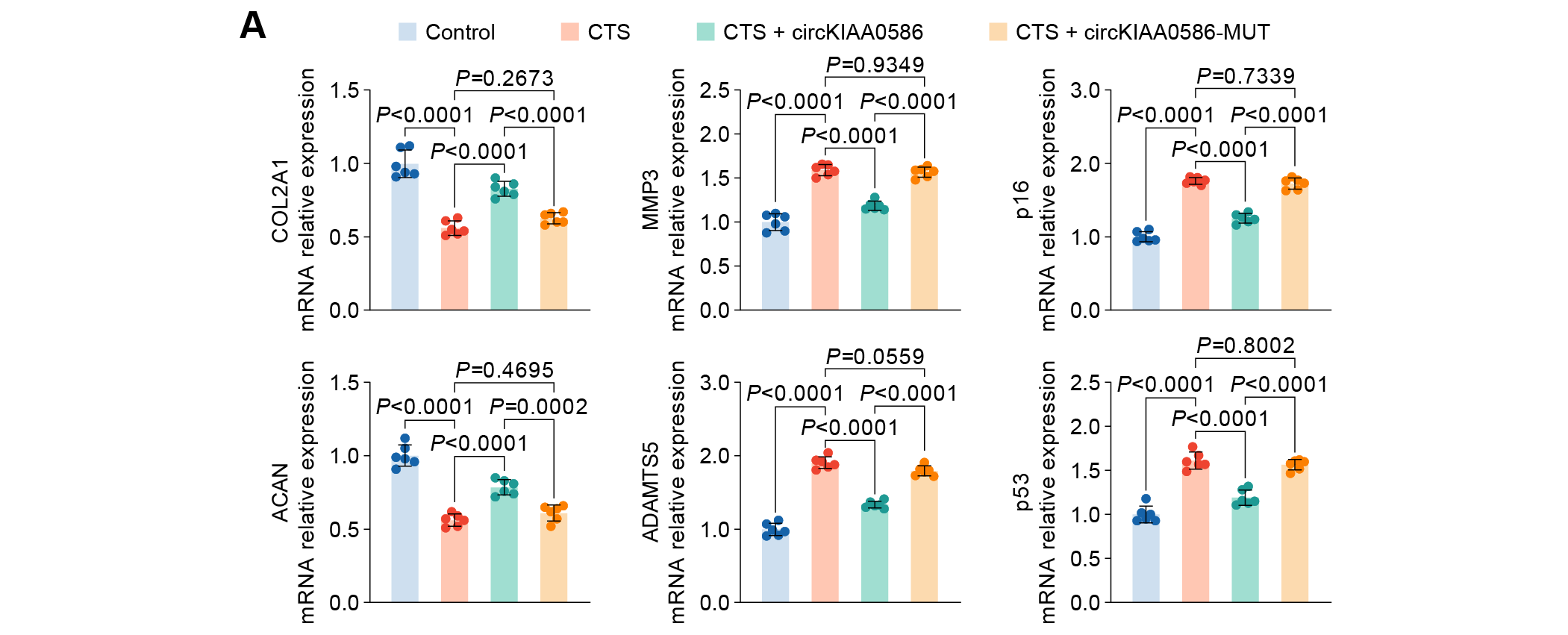


**Figure. S8. CircKIAA0586 alleviates osteoarthritis by targeting miR-335-5p *in vitro*.**

**(A)** Quantitative reverse transcription polymerase chain reaction (qRT-PCR) analysis of COL2A1, MMP3, ACAN, ADAMTS5, and senescence markers (p16 and p53) in controls and human primary chondrocytes treated with 20% cyclic tensile strain (CTS) loading for 24 hours after transfection of control empty vector, circKIAA0586 or circKIAA0586-MUT (*n* = 6 per group).

The statistics are presented as the mean ± standard deviation.


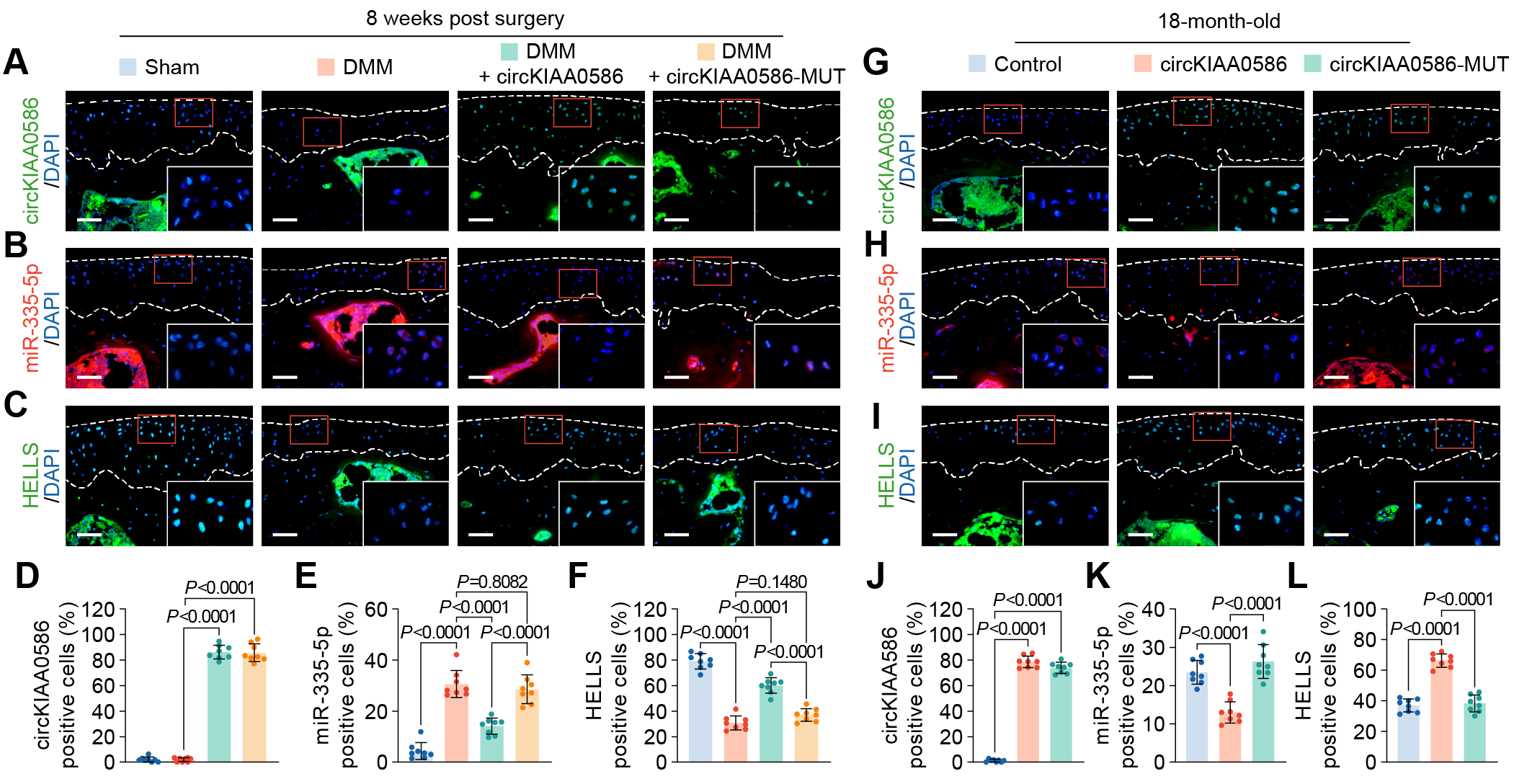


**Figure. S9. CircKIAA0586 regulates miR-335-5p and HELLS *in vivo*.**

**(A**-**F)** Representative images and quantification of circKIAA0586 (A, D) and miR-335-5p (B, E) FISH staining and HELLS immunofluorescence staining (C, F) in knee cartilage from sham mice and destabilization of the medial meniscus (DMM) mice administered control AAV, circKIAA0586 AAV or circKIAA0586-MUT AAV at 8 weeks after DMM surgery (*n* = 8 per group). Scale bar: 40 μm.

**(G**-**L)** Representative images and quantification of circKIAA0586 (G, J) and miR-335-5p (H, K) FISH staining and HELLS immunofluorescence staining (I, L) in knee cartilage from aged spontaneous OA mice administered control AAV, circKIAA0586 AAV or circKIAA0586-MUT AAV (*n* = 8 per group). Scale bar: 40 μm.

All chondrocytes from the surface of cartilage (white dotted line on top) to the boundaries between cartilage and subchondral bone (white dotted line below) were included in the count. Data are presented as the mean ± standard deviation.


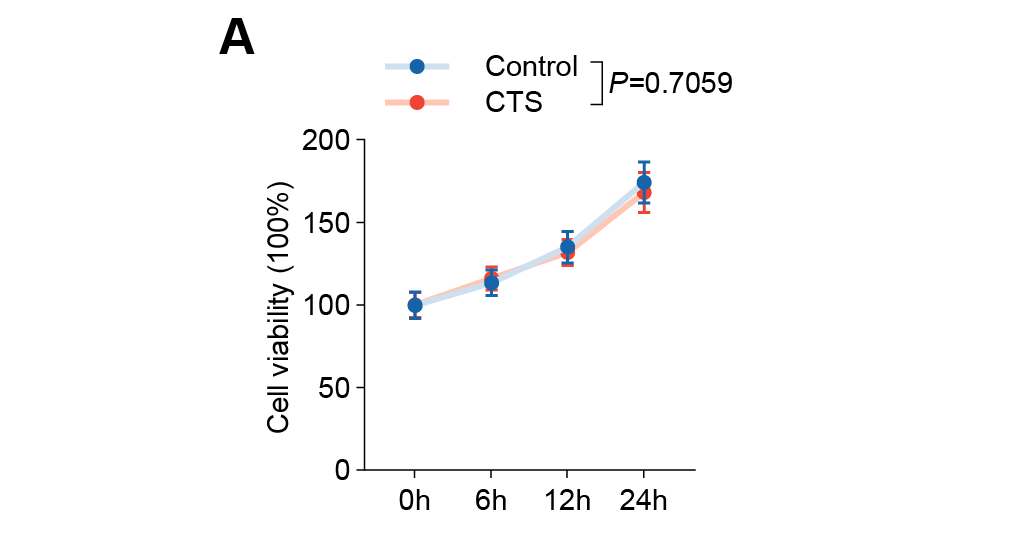


**Figure. S10. Chondrocyte viability is not affected by 20% Cyclic tensile strain at 0.5Hz.**

**(A)** Cell viability of human chondrocytes treated with 20% Cyclic tensile strain at 0.5Hz was detected by CCK-8 assay at 0, 6, 12 and 24 hours.

Data are presented as the mean ± standard deviation.
